# Pressure induced enhancement of the magnetic ordering temperature in rhenium(IV) monomers

**DOI:** 10.1038/ncomms13870

**Published:** 2016-12-21

**Authors:** Christopher H. Woodall, Gavin A. Craig, Alessandro Prescimone, Martin Misek, Joan Cano, Juan Faus, Michael R. Probert, Simon Parsons, Stephen Moggach, José Martínez-Lillo, Mark Murrie, Konstantin V. Kamenev, Euan K. Brechin

**Affiliations:** 1 EaStCHEM School of Chemistry and Centre for Science at Extreme Conditions, The University of Edinburgh, David Brewster Road, Edinburgh EH9 3FJ, UK; 2 School of Engineering and Centre for Science at Extreme Conditions, The University of Edinburgh, Erskine Williamson Building, Peter Gurthrie Tait Road, Edinburgh EH9 3FD,UK; 3 WestCHEM School of Chemistry, University of Glasgow, University Avenue, Glasgow G12 8QQ, UK; 4 School of Physics and Centre for Science at Extreme Conditions, The University of Edinburgh, Erskine Williamson Building, Peter Gurthrie Tait Road, Edinburgh EH9 3FD, UK; 5 Departament de Química Inorgànica/Instituto de Ciencia Molecular (ICMol), Universitat de València, C/ Catedrático José Beltrán no 2, 46980 Paterna (València), Spain; 6 Fundació General de la Universitat de València (FGUV), Universitat de València, Valencia c/Amadeo de Saboya 4, 46010, Spain; 7 School of Chemistry, Newcastle University, Newcastle upon Tyne NE1 7RU, UK

## Abstract

Materials that demonstrate long-range magnetic order are synonymous with information storage and the electronics industry, with the phenomenon commonly associated with metals, metal alloys or metal oxides and sulfides. A lesser known family of magnetically ordered complexes are the monometallic compounds of highly anisotropic d*-*block transition metals; the ‘transformation' from isolated zero-dimensional molecule to ordered, spin-canted, three-dimensional lattice being the result of through-space interactions arising from the combination of large magnetic anisotropy and spin-delocalization from metal to ligand which induces important intermolecular contacts. Here we report the effect of pressure on two such mononuclear rhenium(IV) compounds that exhibit long-range magnetic order under ambient conditions via a spin canting mechanism, with *T*_c_ controlled by the strength of the intermolecular interactions. As these are determined by intermolecular distance, ‘squeezing' the molecules closer together generates remarkable enhancements in ordering temperatures, with a linear dependence of *T*_*c*_ with pressure.

## Abstract (web summary)

Materials that demonstrate long-range magnetic order are synonymous with information storage. Here, the authors report the effect of pressure on two mononuclear rhenium compounds that exhibit long-range magnetic order under ambient conditions via a spin canting mechanism, where *T*_*c*_ is proportional to pressure.

Magnets are ubiquitous in modern society, employed in an enormous range of applications from information storage, biomedical imaging and cancer therapy to space research^1^,^2^,^3^,^4^,^5^. One of the main goals of modern academic and industrial research is device miniaturization, and a bottom–up or molecular approach to building components represents an attractive methodology^6^,^7^. The synthesis of molecules whose behaviour resembles that of classical bulk magnets has been an important challenge for several decades^8^,^9^,^10^,^11^,^12^,^13^,^14^,^15^,^16^,^17^. Here, the physical behaviour is, in part, governed by the magnetic anisotropy of the molecule, which in turn originates from symmetry and structure—factors that are a challenge to control via synthetic chemistry. An alternative way of harnessing and exploiting magnetic anisotropy, and other important factors such as the nature and strength of intra- and intermolecular exchange interactions, is through the use of pressure, since the latter can be used to modify intramolecular bond lengths, angles and metal geometries, and important intermolecular interactions such as H-bonds, C–H···*π* and *π*···*π* contacts, amongst others^18^. Magnetic anisotropy also plays a significant role in spin-canted systems^19^, which behave as weak ferromagnets^10^. In these systems, magnetic order originates from the non-colinearity of neighbouring spin centres which are ‘canted' at a particular angle (*α*) with respect to each other^10^. Importantly the non-negligible intermolecular magnetic interactions can be modified by changing intermolecular distances, for example, making these distances shorter would be expected to increase the strength of the exchange and increase the ordering temperature, *T*_c_ (ref. 20). An obvious way of achieving this is to exert hydrostatic pressure, and by combining high-pressure single-crystal X-ray crystallography and high-pressure SQUID magnetometry the exact relationship between changing structure and changing magnetic behaviour can be extracted^21^.

Among suitable candidates for this type of study are mononuclear complexes based on the hexahalides of the Re^IV^ (5d^3^) ion^20^,^22^,^23^, which are characterised by large magnetic anisotropies and significant intermolecular magnetic exchange^20^. Indeed, such species can display relatively strong dipolar exchange through Re–X···X–Re type contacts (X=halogen) which can result in spin canting^23^,^24^,^25^,^26^,^27^. DFT calculations reveal these exchange pathways arise as a result of the spin density from the metal ion being delocalized onto the peripheral atoms of the ligands^28^,^29^,^30^, an effect not observed in analogous 3d metal complexes which do not order. Herein we report a high pressure study of the complexes [ReCl_4_(MeCN)_2_]·MeCN (**1**) and [ReBr_4_(bpym)] (**2**) (bpym=2,2′-bipyrimidine). **1** is a well-known starting material for the preparation of rhenium-based compounds. Curiously, although a straightforward synthetic method to **1** has been known since 1968 (ref. 30), and its crystal structure was recently solved^31^,^32^, its magnetic behaviour has never been reported. The synthesis and crystal structure of **2** (ref. 33), together with electrochemical^33^ and preliminary magnetic studies^25^, are known. **1** and **2** exhibit magnetic ordering via spin canting. Here we demonstrate that a rather remarkable enhancement of *T*_c_ can be achieved for both **1** and **2** through the application of modest pressures. A combined high-pressure structural and magnetic study reveals that shortening intermolecular interactions result in a linear dependence of *T*_c_ with pressure, and a complementary theoretical study extracts the changes in both the exchange interaction parameters and anisotropy of the Re^IV^ ion that lead to this enhancement.

## Results

### High-pressure X-ray diffraction studies of 1 and 2

**Figure 1:**
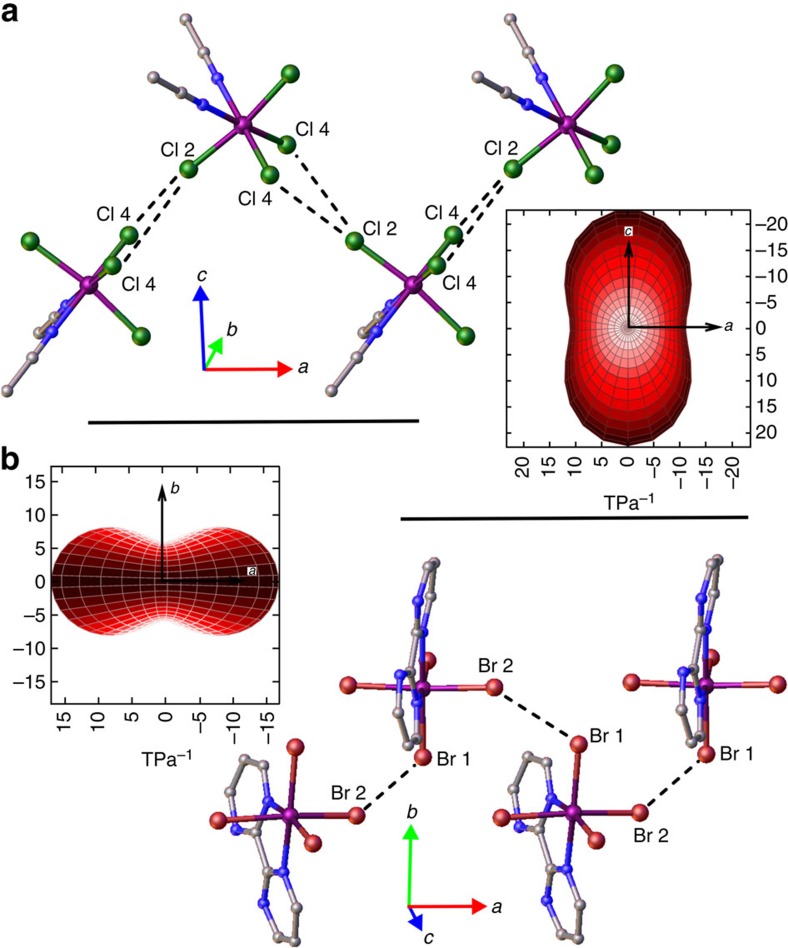
Structural comparison of 1 and 2 at high pressure. Comparison of the packing between chains showing the shortest intermolecular Re–X··X–Re interactions in **1** (**a**) and **2** (**b**). Inserts: compressibility indicatrix of **1** and **2** generated from the high-pressure data showing the spatial orientation of strong-positive compressibility axes relative to their crystallographic axes and chain direction. In **1** the principal axis of compression lies perpendicular to the Re–X···X–Re chain, while in **2** it appears parallel to the equivalent interactions. The disceprancy in behaviour is attributed to the continuous void occupied with MeCN that runs along the *c* axis in **1**.

The crystal structures of compounds **1** and **2** at ambient pressure have been reported and discussed elsewhere^31^,^32^,^33^. However, it is useful to discuss certain features to understand the structural changes observed with hydrostatic pressure, and the possible effects that such changes may have on the magnetic properties. Both compounds crystallise in orthorhombic space groups (*Pnma* and *P*2_1_2_1_2_1_ for **1** and **2**, respectively) and, although not isostructural, they have several features in common that may aid the explanation of the unique magnetic behaviour observed at pressure. Both compounds exhibit a Re^IV^ ion in a somewhat distorted octahedral environment, being bonded by two N atoms coordinated *via* the organic ligand(s) and four halide anions (Supplementary Fig. 1). Both display staggered zig-zag chains that propagate along the *a* axis of the unit cell via interhalide dispersive interactions (Fig. 1), with **1** forming a layered structure of chains of [ReCl_4_(MeCN)_2_] alternating with disordered MeCN molecules that occupy voids that run parallel to the chains. The chains observed in **2** pack to form sheets in a manner reminiscent of herring bone type structures (Supplementary Fig. 2).

On compression, the structures of **1** and **2** undergo significant contraction, reflected in a reduction in cell parameters and volume for both (Supplementary Tables 1 and 2, and Supplementary Data 1 and 2). The most noticeable effect is the reduction of the unit cell volume from 1381.11(8) Å^3^ under ambient conditions to 1102.4(13) Å^3^ at 4.30 GPa—a change of 20% for **1**, and from 1402.5 Å^3^ at ambient to 1202.3 Å^3^ at 3.64 GPa—a change of 14% for **2**. In both systems the principal component of compression occurs down the *c* axis of the unit cell, and the contraction occurs in a way that compresses the structure into the voids occupied by the disordered solvent in **1** (Fig. 1 and Supplementary Table 3). An important point to note is the structural transformation of compound **2** between 1.93 GPa and 3.06 GPa from the ambient *P*2_1_2_1_2_1_ orthorhombic phase to a previously unreported monoclinic phase with space group *P*2_1_ (Supplementary Data 2). The transformation results in two molecules of **2** in the asymmetric unit opposed to the one molecule observed in the ambient phase.

The behaviour of the intermolecular Re–X···X–Re distances is of great importance in these systems^20^. Under ambient conditions there are two types of halogen–halogen interactions in **1** of very similar distances (Fig. 1 and Supplementary Table 4); Re–Cl(4)···Cl(4)–Re is 4.014(2) Å and Re–Cl(4)···Cl(2)–Re is 3.9217(13) Å. While the application of pressure does not seem to affect the former very much (3.912(14) Å at 4.30 GPa), the latter shortens by ∼0.6 Å to 3.362(11) Å. Compound **2** displays a similar reduction in interbromide distances, albeit smaller on average, with Re-Br(2)···Br(4)-Re changing by 0.31 Å from ambient to 3.64 GPa and reducing in a manner that is reflective of the more isotropic compression of the material (Supplementary Table 5).

Analysis of the significant intramolecular bond lengths for **1** and **2** reveals limited change with pressure for both compounds. The values of the Re–Cl and Re–N bond lengths in **1** are in agreement with those previously reported and they remain essentially constant with the different applied pressures (Supplementary Table 6). Similar behaviour is observed in **2** (Supplementary Table 7). However, the axial Cl(2)–Re(1)–Cl(3) angle distorts significantly from ‘linearity' with increasing pressure (Supplementary Fig. 3) by ∼3.0° (**1**) and 4.3° (**2**) from that observed under ambient conditions, resulting in a significant distortion to the previously discussed octahedron. This distortion may have important consequences for the anisotropy of the Re^IV^ ion (*vide infra*).

Both samples were subjected to complete compression-decompression cycles up to 4 GPa and were found to return to their original ambient pressure unit cell and space group with no significant changes in structure. To exclude the possibility that a low temperature structural transition drives modulation of the magnetic properties in **1** and **2** (*vide infra*), their crystal structures were solved at *T*=4 K. On cooling, both materials were found to retain their room temperature structures, with the only differences arising from the expected thermal contraction (Supplementary Table 8 and Supplementary Fig. 4).

### High-pressure magnetic studies of 1 and 2

**Figure 2:**
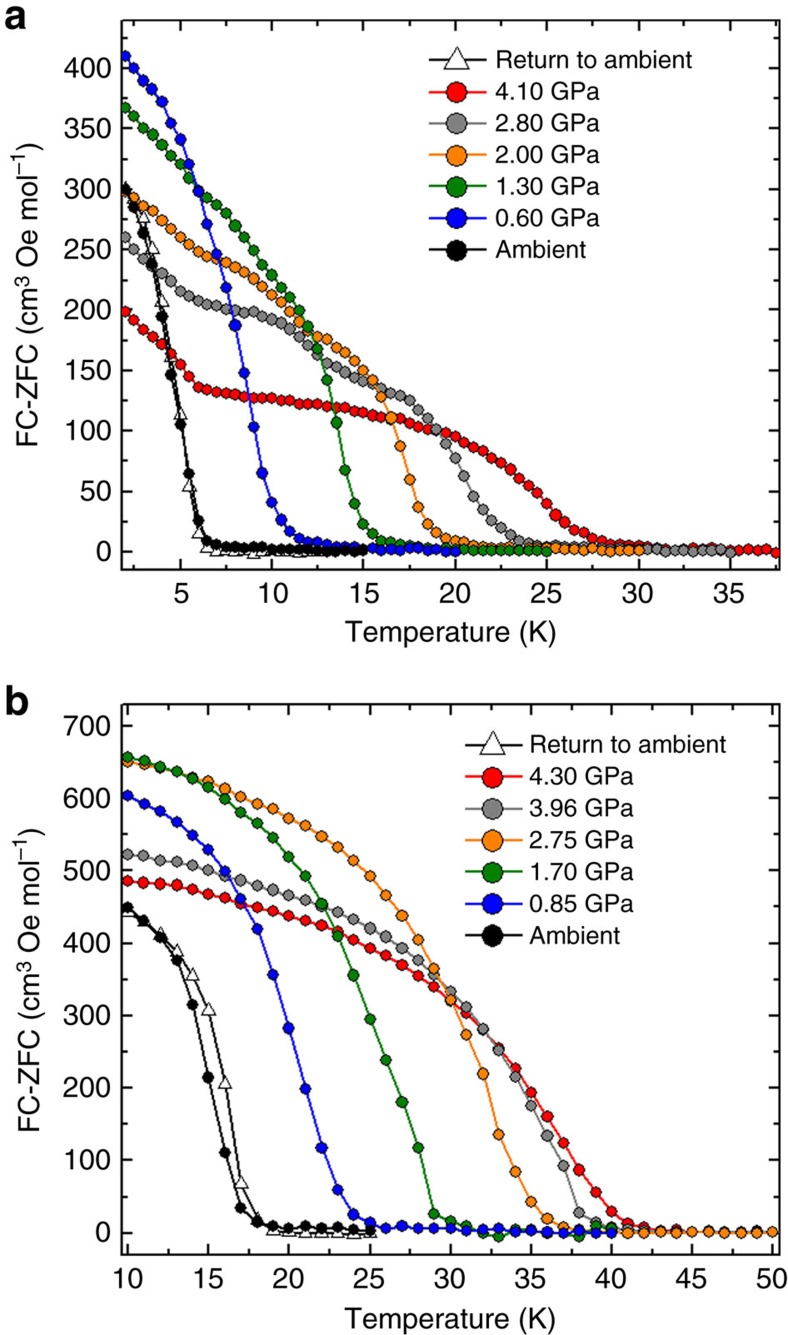
Pressure dependence of the magnetic properties of 1 and 2. Plot of field cooled−zero-field cooled curves versus temperature for **1** (**a**) and **2** (**b**). In both compounds the *T*_c_ value is shifted to higher temperatures with increasing pressure. Both compounds were measured under an applied field of 100 Oe. High-pressure data were collected in a turnbuckle DAC designed specifically for use in an MPMS magnetometer, while the return to ambient pressure were measured on the same samples in a gelatine caspule.

**Figure 3:**
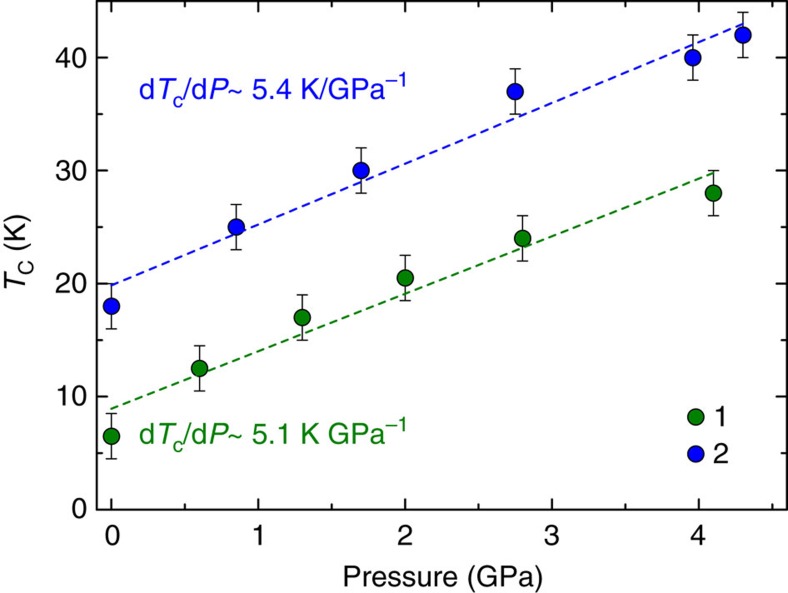
Enhancement rates of the magnetic ordering temperature for compounds 1 and 2. The magnetic ordering temperature (*T*_C_) versus applied pressure plot for **1** (green) and **2** (blue) shows a linear increase of *T*_C_ values with applied pressure. For **1** this corresponds to 5.1 K GPa^−1^, and for **2** this corresponds to 5.4 K GPa^−1^. The error bars for each data point represent twice the size of the temperature measurement interval.

To determine the impact that the pressure-induced structural modifications have on the magnetic properties of compounds **1** and **2**, HP SQUID magnetometry was employed. An ambient pressure magnetic study of **1** as measured on a sample in a gelatine capsule is given in Supplementary Note 1 and Supplementary Figs 5 and 6. High-pressure dc magnetic susceptibility measurements were performed on microcrystalline samples of **1** and **2** in a turnbuckle diamond anvil cell^34^. The ordering temperature for both compounds, *T*_C_, was determined by field cooled and zero-field cooled measurements carried out under a field of 100 Oe, the temperature at which the remanent magnetization becomes non-zero. Figure 2 shows the field cooled–zero-field cooled curves as a function of temperature for compounds **1** and **2** at the various pressure points measured. At ambient pressure in the turnbuckle cell, compounds **1** and **2** display *T*_C_ values of 6.5 and 18.0 K, respectively. For both complexes, the application of hydrostatic pressure leads to an increase in the size of the remanent magnetization measured, a phenomenon that was ascribed to the emergence of anisotropy in the case of the compound Mn[N(CN)_2_]_2_ (ref. 35). More remarkable, however, is the rise observed in the ordering temperature of **1** and **2** as the pressure is increased. At the highest pressures measured, *T*_C_ reaches a value of 28 K at 4.10 GPa for compound **1**, and 42 K at 4.30 GPa for compound **2**. In the case of compound **1**, this represents a four-fold enhancement of the ordering temperature. The rate at which *T*_C_ increases with pressure was determined to be ∼5.1 and 5.4 K GPa^−1^ for **1** and **2**, respectively (Fig. 3). The phase transition observed for compound **2** above 1.93 GPa does not appear to affect the monotonic increase of the ordering temperature. These increases in *T*_C_ are consistent with the contraction in the intermolecular halogen···halogen distances revealed by the high-pressure crystallographic study. To demonstrate that the effects of pressure on the magnetic properties of **1** and **2** are reversible, the pressure was released from the cell, the samples removed and their remanent magnetization measured in gelatine caspules. In both cases, a recovery of the ambient pressure *T*_C_ is observed (Fig. 2). To analyse the exchange interactions in a quantitative manner, and to evaluate a possible change in the magnetic anisotropy of the distorted octahedral Re^IV^ ion, we now turn to theory.

### Theoretical studies

**Figure 4:**
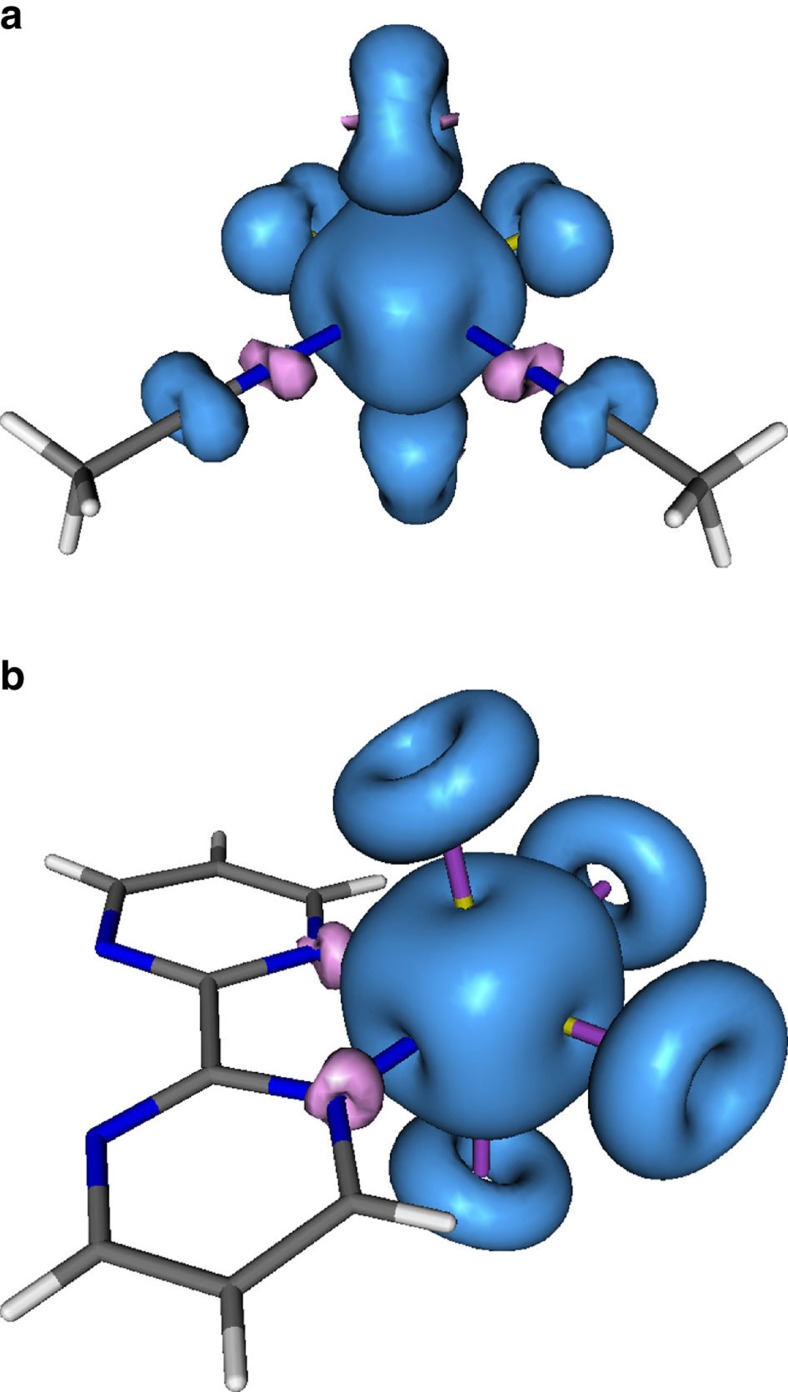
Spin densities calculated through DF for 1 and 2. View of the calculated spin density for the *S*=3/2 ground spin configuration of the [ReCl_4_(MeCN)_2_] (**a**) and the [ReBr_4_(bipyrimidine)] (**b**) complexes in **1** and **2**, respectively. The isodensity surface corresponds to a cutoff value of 0.003 e bohr^−3^. Blue and magenta isosurfaces correspond to positive and negative regions of spin density, respectively. Spin densities are largely centred on the metal ion, but with strong delocalization to the chloride and bromide ions. Non-negligible spin polarization (magenta) is observed on the nitrogen atoms of the N-donor ligands.

The first question to be answered by theory is if the necessary conditions to observe spin canting have been met, and thus we have employed density functional (DF) calculations to evaluate the magnetic exchange interactions between Re^IV^ ions, and the evolution of this exchange with pressure. The only magnetic interactions that can exist in **1** and **2** are intermolecular in nature. Dipolar magnetic exchange is normally rather weak, and indeed for 3d metals even negligible. However, this is not the case for mononuclear Re^IV^ complexes in which there exists strong *π* spin delocalization from the Re^IV^ ion onto the coordinated ligands, particularly to the donor atoms that are directly connected to the metal ion. The result is non-negligible magnetic interactions between spin densities on neighbouring molecules^20^. Figure 4 shows spin density maps for **1** and **2** which reveal large spin delocalization onto the chloride **1** and bromide **2** ions, and little to the peripheral regions of the organic ligands. Significant spin polarization is also seen on the nitrogen atoms of the acetonitrile and bipyrimidine molecules that are directly coordinated to the metal ion in **1** and **2**, respectively. The important intermolecular interactions would be expected to occur only between atoms exhibiting large spin densities.

**Figure 5:**
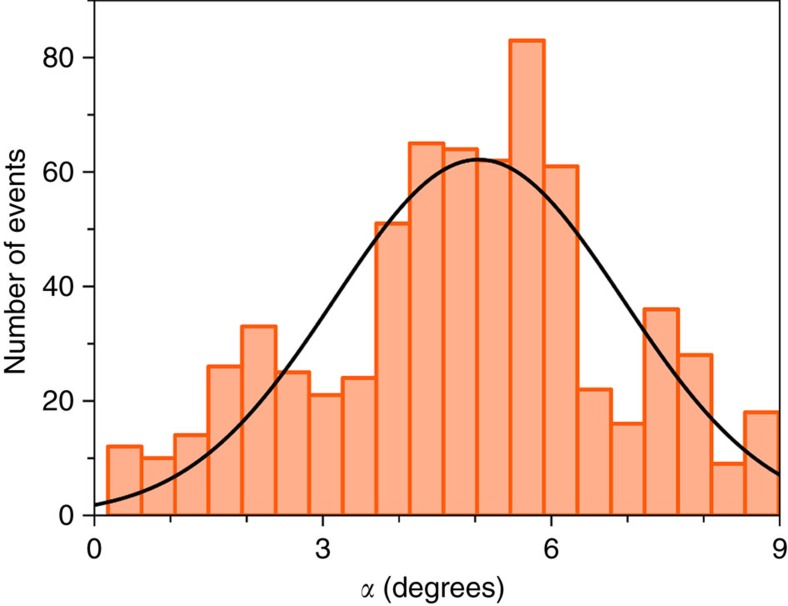
Histogram of the *α* value in 1. Distribution of the *α* value obtained from molecular dynamics at *T*=5 K. Bars show the number of events for each interval value and the solid black line is the best-fit to a Gaussian curve. A total of 1,000 events were recorded with a time length of 1 fs per step. A maximum in the distribution is observed around *α*=5.0°.

Several intermolecular contacts were selected after a detailed inspection of the crystal packing of both compounds (Supplementary Figs 7 and 8) and DF calculations were performed on these moieties to estimate the magnitude and nature of the magnetic exchange (*J*_i_) at varying pressures. The computed *J*_i_ values are given in Supplementary Tables 9 and 10. As expected, these results show that the shortest pathways involving the chloride and bromide anions generate the most efficient magnetic exchange at all pressures (*J*_1–3_ in **1** and *J*_1–2_ in **2**). The magnitude of these interactions is indeed large enough to induce magnetic order. However for spin canting the easy-axis of magnetization of the exchange coupled metal centres must also be strictly non-colinear. Several factors can contribute to controlling the orientation of these axes, but there is little doubt that the presence of magnetic anisotropy is crucial. The axial (*D*) and rhombic (*E*) zero-field splitting (zfs) parameters for **1** and **2** were evaluated from CAS calculations, confirming very large axial magnetic anisotropy arising from second-order spin–orbit coupling (Supplementary Table 11). From these results it is also possible to determine *D* tensor projections and the angles (*α*) between projections on different Re^IV^ ions (Supplementary Fig. 9). We can then compare the theoretically derived *α* angles (*α*_THEO_=0 and 7.3°) with those found experimentally (*α*=2.0 and 2.5° for **1** and **2**, respectively; see Supplementary Note 1) from the saturation value of the magnetization, or from the saturated magnetic susceptibility at the lowest temperature^25^. In **2**, the strongest magnetic exchange (*J*_1_=–2.25 cm^–1^) leads to a theoretical *α*_THEO_=7.3°, formed by the *z* components of the *D* tensor of two neighbouring [ReBr_4_(bpym)] complexes, close to that extrapolated from experiment, and confirming non-colinearity. The situation in **1** is less straightforward since at ambient pressure three similar intermolecular magnetic interactions coexist, and inversion symmetry between neighbours should strictly define *α*=0. Experimentally, the presence of a non-zero *α* angle can originate from several sources, including non-isotropic exchange interactions and/or a difference in the structure/symmetry of the complex at high temperature where the X-ray structure was solved and low temperature where the magnetic order occurs. However, low temperature (*T*=4 K) single-crystal X-ray diffraction at ambient pressure shows no significant change to the structure in **1** or **2**, ruling out the latter. To visualize if a non-zero value of the *α* angle can exist, a molecular dynamics simulation of **1** was performed with DF methods in the solid state at *T*=5 K from a pre-optimized structure. From NEVPT2 and CAS methods, an evaluation of the zfs parameters, including the orientation of the *D* tensor, was made on all the geometries found in the molecular dynamics of the two neighbouring [ReCl_4_(MeCN)_2_] units (**a** and **b**) involved in the *J*_1_ magnetic exchange. The results are shown in Fig. 5 and Supplementary Figs 10 and 11. Very similar results were found from both NEVPT2 and CAS calculations, revealing non-negligible changes in the time evolution of the *α* angle and *D* parameter for **a** and **b**.

Histograms of the time evolutions show that the values of both parameters follow a Gaussian distribution centred at *α*_SIM_=5.0° and *D*_SIM_=–26.0 cm^–1^. The *D*_SIM_ value is very close to that obtained theoretically from the experimental crystal structure at ambient pressure (*D*_THEO_=–30.0 cm^–1^) and also to that obtained experimentally from the fit of *χ*_M_*T versus T* plot in the temperature range 20–300 K (*D*_EXP_=–37.1 cm^–1^; Supplementary Fig. 5). The non-zero *α*_SIM_ value supports the observed spin canting phenomenon in **1**. Supplementary Movies 1 and 2 allow us to visualize the changes in the geometries of **a** and **b** with time. Although these structural changes are not large, they are big enough to induce modifications in the *D* parameter, and significant enough to allow the spin-canting phenomenon to occur. In short, the significant zfs allows the magnetization axes on neighbouring metal centres to be non-collinear, resulting in spin canting.

Anisotropy calculations were performed on the molecular geometries of **1** and **2** at higher pressures (Supplementary Note 2), and a summary of the results is given in Supplementary Table 11 and Supplementary Figs 12 and 13. Compound **2** is magnetically more anisotropic when external pressure is applied, whereas **1** becomes less anisotropic. However, the changes are rather small in both cases. The changes in the *D* parameter are easily explained by changes to the molecular geometry, and in particular to distortions in the axial X–Re–X moiety. Only the spin–orbit part is significant here, with contributions from both the quartet and doublet excited spin states, the latter being the most important (Supplementary Note 2). Thus, changes to the octahedral geometries of the Re ions in **1** and **2** with pressure have changed their electronic structure energy diagrams, that is, the relative energies of the excited states have been modified and their contributions to the zfs have changed accordingly. However, the observed distortions are similar (and relatively small) in both complexes [3.0 (**1**) and 4.3° (**2**)] and the modification of *D* in each case is not enough to explain the large changes in *T*_c_.

**Figure 6:**
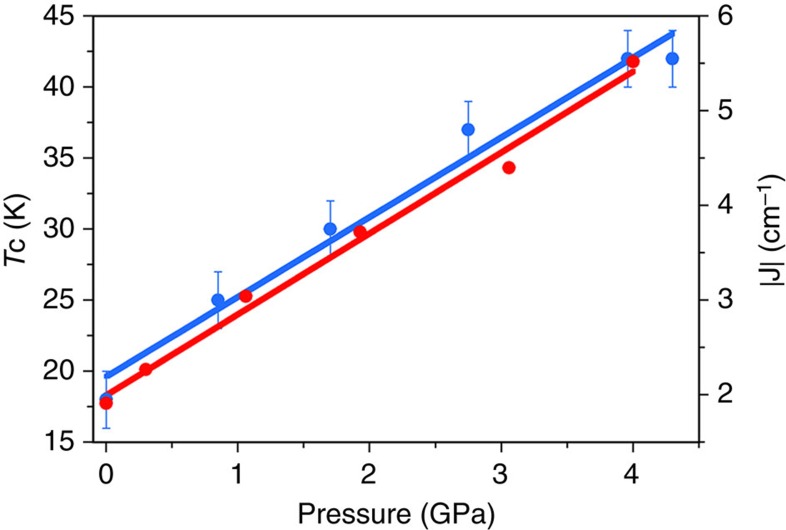
Magnetostructural correlations in 2. Pressure dependence of the ordering temperature, *T*_c_ (blue circles), and the strongest magnetic exchange, *J* (red circles). The lines represent the linear best-fit. The magnetic exchange interactions were calculated on the experimental geometries for each experimental pressure determined. The data points above 3.5 GPa are above the pressure where the structural phase transition is seen crystallographically. The error bars for each data point of the experimentally derived *T*_c_ are given.

On the other hand, significant changes in the magnitude of the intermolecular magnetic exchange interactions can considerably influence the magnetic behaviour. Thus, an increase in *T*_c_ would be expected when the magnetic exchange constants become stronger. DF calculations reveal an increase in the magnitude of the *J*_i_ parameters in **1** and **2** when external pressure is applied, in agreement with increasing *T*_c_ in both systems. In **2**, the correlation between parameters is beautifully straightforward, external applied pressure produces a linear increase in *J*_1_ and a linear increase in *T*_c_ (Fig. 6). The analysis of **1** is again complicated by the presence of three similar *J*_i_ values, although the overall trend seen above is repeated (Supplementary Fig. 14), and only the *J*_i_ value at 4.3 GPa deviates from the expected correlation. We focus on the *J*_1_ interaction. In **2** the *J*_1_ magnetic exchange only involves the spin densities on neighbouring Br ions, the distance between which shortens with pressure. In **1**, *J*_1_ again involves the halide···halide contact but also that between halide and both the N and C atoms of an acetonitrile molecule. In other words, three contacts are directly involved in the interaction. To complicate matters further, the spin densities on the C and N atoms exhibit different signs, and moreover, the intermolecular distances in these three contacts do not vary in the same manner with pressure (Supplementary Fig. 15). Thus, deconvoluting the individual contributions of these three simultaneous changes is non-trivial. From Supplementary Fig. 14 it is clear that the *T*_c_ and *J* values calculated for the highest pressure measured (4.3 GPa) deviate from the linear correlation. This can be attributed to the anomalously large carbon–nitrogen bond lengths in the coordinated acetonitrile molecule that are clearly very different to the values found at all other pressures (Supplementary Tables 12 and 13). This structural discrepancy is associated to the difficulties in obtaining structural data at such high pressures. The result is an underestimation of the strength of the magnetic exchange at this pressure.

## Discussion

While chemists have become accustomed to the use of high temperatures and high magnetic fields, the use of high-pressure techniques remains hugely under-exploited—particularly in molecular chemistry. This is surprising given that the physical properties of molecules are inherently linked to their structures and any modification of the latter will have important, and potentially game-changing consequences on the former. Perhaps one reason is the oft-heard but incorrect assumption that the only consequence of applying pressure to molecules in crystals is the removal of interstitial void space. This is the obvious initial consequence, but when these voids are removed the molecules push against each other and it is at this point and beyond where novel physical properties emerge. Here, we have demonstrated the pressure-induced enhancement of the magnetic ordering temperatures of two spin-canted Re^IV^ systems, and have correlated changes in the magnetic behaviour with changes in structure through the combined use of high-pressure single-crystal X-ray crystallography and high-pressure SQUID magnetometry. This high-pressure methodology has allowed unprecedented insight into the phenomenon in a step-by-step manner, observing remarkable reductions in unit cell volume, with the concomitant shortening of intermolecular distances resulting in a linear increase in *T*_c_. DF-type calculations have allowed us to evaluate the magnetic exchange interactions (*J*_i_) between the Re^IV^ ions, and how these *J*_i_ values are affected by the application of pressure. The *J*_i_ values increase with increasing pressure on account of closer intermolecular contacts enforcing stronger exchange coupling between adjacent Re^IV^ centres at each increased pressure. Pressure-induced enhancement of *T*_c_ has been previously reported through SQUID magnetometry in 3D ferro- and ferrimagnets, and in weak ferromagnets containing transition metal ions^35^,^36^,^37^,^38^,^39^,^40^,^41^,^42^,^43^, although in each case no structural information was forthcoming, preventing unequivocal explanation of the observed magnetic changes. The present study represents the first combined high-pressure single-crystal X-ray crystallography—high-pressure magnetism study of an ordered 5d molecule-based material. The changes in *T*_c_ are quite remarkable and originate, in the main, from a simple shortening of intermolecular distances, which results in larger dipolar magnetic exchange. This opens the gateway to studying the pressure-dependent magnetic behaviour of the relatively large number of anisotropic, monomeric transition metal complexes which also order at low temperatures. One can imagine similarly impressive results in such systems, particularly for 5d metal ions on account of their increased magnetic anisotropy arising from their substantial spin–orbit coupling constants and the larger diffuseness of their magnetic orbitals when compared with those of the 3d and 4d ions^44^. Indeed the linear increase in *T*_c_ with pressure seen here suggests that enhancement rates in other species may be much larger and that pressure could be employed to invoke transition from paramagnetism to LRMO.

Finally, we hope to have demonstrated the power, usefulness and potential of employing high-pressure techniques in molecular chemistry. Here we employed high-pressure X-ray crystallography and high-pressure SQUID magnetometry to reveal the relationship between structure and magnetic behaviour, but other high-pressure techniques also exist and remain largely unexplored. For example, high-pressure INS, Raman, infrared/ultraviolet–visible/near-infrared and EPR spectroscopies are all available and offer the chemist a wonderful palette of high-pressure techniques with which to investigate the physical properties of molecules. In combination this proffers an extremely powerful toolkit.

## Methods

### Synthesis of **1** and **2**

All manipulations were performed under aerobic conditions, using chemicals as received. Type 3 Å molecular sieves were used to dry the MeCN before use. Compound **1** was prepared following the literature procedure^32^. The synthesis of **2** was performed following an alternative method to that described in the literature and starting from (NBu_4_)_2_[ReBr_6_], which was previously prepared by a metathesis reaction of K_2_ReBr_6_ in a 0.5 M HBr solution^33^. A mixture of (NBu_4_)_2_[ReBr_6_] (0.15 g, 0.13 mmol) and 2,2′-bipyrimidine (0.02 g, 0.13 mmol) in 5 ml of glacial acetic acid was heated at 90 °C with continuous stirring for 2 h. It was then filtered and the obtained red–orange solution was left to evaporate in a fumehood at room temperature. Dark red crystals of **2** suitable for X-ray diffraction studies were formed in 2 days. Yield: 40%. Analysis {calcd., found for C_8_H_6_N_4_Br_4_Re (**2**)}: C (14.5, 14.3), H (0.9, 1.1), N (8.4, 8.5) %. IR peaks (KBr/cm^−1^): 1,576(vs), 1,544(m), 1,404(vs), 814(m), 743(s), 665(m) for **2**.

### Structure determination and refinement

Single-crystal X-ray diffraction data and high-pressure data were collected on a Bruker APEX II diffractometer with graphite-monochromated Mo–K*α* radiation with a wavelength of 0.71073 Å for both compounds **1** and **2**. The high-pressure series collected on compound **1** was collected at station I19 at Diamond Light Source, using radiation of wavelength 0.48590 Å on a four-circle Crystal Logic diffractometer equipped with a Rigaku Saturn CCD detector. A Merrill–Bassett diamond anvil cell (half-opening angle 40°), equipped with Boehler–Almax diamonds with 600 μm culets and a tungsten gasket was used^45^. Pressure was measured before and after each data collection via ruby fluorescence, with compound **1** loaded in a 40:60 petroleum ether mixture and compound **2** in Daphne 7373 oil^46^. Cell indexing and data processing were carried out using the Bruker APEX II suite, with the Rigaku frames obtained in I19 being converted to Bruker compatible frames using the programme ECLIPSE^47^. Integration was carried out using SAINT, with dynamic masks generated by ECLIPSE. Absorption corrections were carried with SHADE, to account for cell shading and with the routine non-empirical method of SADABS^48^. Structure solution and space group determination were carried out using SHELXT^49^ for the new phase of compound **2**. The ambient in-cell data for compound **2** were collected after the pressure series. On decompression the crystal split and was integrated and refined as a twinned crystal. All other structures were modelled from the starting coordinates. Refinements were carried out for compound **1** using CRYSTALS (against *F* using reflections with I>2*σ*) and SHELXL through the Olex2 software suite for compound **2** (against *F*^2^ using reflections with I>2*σ*)^50^. All metal-ligand distances and angles, and all torsion angles were refined freely, with RIGU^51^ thermal and vibrational similarity restraints applied throughout. The compressive indicatrices were calculated using Pascal^52^.

### Magnetic studies

Dc magnetic susceptibility and magnetization measurements at ambient pressure were carried out on a microcrystalline sample of **1** in a Magnetic Property Measurement System (MPMS, Quantum Design, USA) magnetometer equipped with a 7 T magnet operating in the 300–1.9 K temperature range. High-pressure dc magnetic susceptibility measurements were carried out in an MPMS SQUID equipped with a 5 T magnet on microcrystalline samples (∼10 μg) of **1** and **2** (**1** @ ambient—4.1 GPa; **2** @ ambient—4.2 GPa) in the 50.0–2.0 K temperature range and an external magnetic field of 100 Oe in a turnbuckle diamond anvil cell with 800 μm culets, using ruby powder as the pressure calibrant, and Daphne 7,373 oil as the pressure transmitting medium. The cell and gasket were made from ultrapure CuBe 165. See ref. 34 for full details of the cell.

### Computational methods

Full details of the computational methodology are given in the Supplementary Methods and Supplementary References.

### Data availability

All data supporting the findings of this study are available from the authors. The X-ray crystallographic data (CIF files) for the structures reported in this work have been deposited at the Cambridge Crystallographic Data Centre (CCDC), under deposition numbers CCDC 966619–966625 (**1**), CCDC 1453399–1453406 (**2**) and CCDC 1491360–1491361 (ambient, *T*=4 K for **1** and **2**). These data can be obtained free of charge from The Cambridge Crystallographic Data Centre via www.ccdc.cam.ac.uk/data_request/cif. All other data may be obtained from Edinburgh Datashare: http://datashare.is.ed.ac.uk/. Alternatively, data may be obtained from the corresponding authors upon request.

## Additional information

**How to cite this article:** Woodall, C. H. *et al*. Pressure induced enhancement of the magnetic ordering temperature in rhenium(IV) monomers. *Nat. Commun.* **7,** 13870 doi: 10.1038/ncomms13870 (2016).

**Publisher's note:** Springer Nature remains neutral with regard to jurisdictional claims in published maps and institutional affiliations.

## Supplementary Material

Supplementary Information Supplementary Figures, Supplementary Tables, Supplementary Notes, Supplementary Methods and Supplementary References.

Supplementary Data 1 Crystallographic files for compound 1 at various pressures and temperatures.

Supplementary Data 2 Crystallographic files for compound 2 at various pressures and temperatures.

Supplementary Movie 1 a view of the two [ReCl4(MeCN)2] moieties involved in the J1 interaction in compound 1. Specifically it shows a dynamic visualisation at T = 5 K of the relative placement of two [ReCl4(MeCN)2] molecules mediating the J1 magnetic exchange, viewed direction along the [010] lattice vector.

Supplementary Movie 2 a view of two [ReCl4(MeCN)2] moieties involved in the J1 interaction in 1. Specifically it shows a dynamic visualisation at T = 5 K of the relative placement of two [ReCl4(MeCN)2] molecules mediating the J1 magnetic exchange, viewed along the [101] lattice vector.

Peer Review File

## References

[b1] Cho M. H. . A magnetic switch for the control of cell death signalling *in vitro* and *in vivo* systems. Nat. Mater. 11, 1038–1043 (2012). 23042417 10.1038/nmat3430

[b2] Pankhurst Q. A., Connolly J., Jones S. K. & Dobson J. Application of magnetic nanoparticles in biomedicine. J. Phys. D 36, R167–R181 (2003).

[b3] Dobson J. Remote control of cellular behavior with magnetic nanoparticles. Nat. Nanotechnol. 3, 139–143 (2008). 18654485 10.1038/nnano.2008.39

[b4] Schenck J. F. Safety of strong, static magnetic fields. J. Magn. Reson. Imaging 12, 2–19 (2000). 10931560 10.1002/1522-2586(200007)12:1<2::aid-jmri2>3.0.co;2-v

[b5] Díaz-Michelena M. Small magnetic sensors for space applications. Sensors 9, 2271–2288 (2009). 22574012 10.3390/s90402271 PMC3348798

[b6] Mannini M. . Magnetic memory of a single-molecule quantum magnet wired to a gold surface. Nat. Mater. 8, 194–197 (2009). 19182788 10.1038/nmat2374

[b7] Tetienne J.-P. . Nanoscale imaging and control of domain-wall hopping with a nitrogen-vacancy center microscope. Science 344, 1366–1369 (2014). 24948732 10.1126/science.1250113

[b8] Miller J. S. & Gatteschi D. Molecule-based magnets. Chem. Soc. Rev. 40, 3065–3066 (2011). 21552607 10.1039/c1cs90019f

[b9] Miller J. S. Organic- and molecule-based magnets. Mater. Today 17, 224–235 (2014).

[b10] Kahn O. Molecular Magnetism VCH (1993).

[b11] Sessoli R., Gatteschi D., Caneschi A. & Novak M. A. Magnetic bistability in a metal-ion cluster. Nature 365, 141–143 (1993).

[b12] Wernsdorfer W. & Sessoli R. Quantum phase interference and parity effects in magnetic molecular clusters. Science 284, 133–135 (1999). 10102810 10.1126/science.284.5411.133

[b13] Leuenberger M. N. & Loss D. Quantum computing in molecular magnets. Nature 410, 789–793 (2001). 11298441 10.1038/35071024

[b14] Wernsdorfer W., Aliaga-Acalde N., Hendrickson D. N. & Christou G. Exchange-biased quantum tunnelling in a supramolecular dimer of single-molecule magnets. Nature 416, 406–409 (2002). 11919625 10.1038/416406a

[b15] Aromí G. & Brechin E. K. Synthesis of 3d metallic single-molecule magnets. Struct. Bonding 122, 1–69 (2006).

[b16] Craig G. A. & Murrie M. 3d single-ion magnets. Chem. Soc. Rev. 44, 2135–2147 (2015). 25716220 10.1039/c4cs00439f

[b17] Martínez-Lillo J. . Highly anisotropic rhenium(IV) complexes: new examples of mononuclear single-molecule magnets. J. Am. Chem. Soc. 135, 13737–13748 (2013). 23957361 10.1021/ja403154z

[b18] Prescimone A. . [Mn_6_] under pressure: a combined crystallographic and magnetic study. Angew. Chem. Int. Ed. 47, 2828–2831 (2008). 10.1002/anie.200705819 18324653

[b19] Wang X.-Y., Avendaño C. & Dunbar K. R. Molecular magnetic materials based on 4d and 5d transition metals. Chem. Soc. Rev. 40, 3213–3238 (2011). 21409195 10.1039/c0cs00188k

[b20] Martínez-Lillo J., Faus J., Lloret F. & Julve M. Towards multifunctional magnetic systems through molecular-programmed self assembly of Re(IV) metalloligands. Coord. Chem. Rev. 289−290, 215–237 (2015).

[b21] Awaga K. & Maruyama Y. High-pressure effects on the canted ferromagnetism in manganese(II) phthalocyanine. Phys. Rev. B 44, 2589–2594 (1991). 10.1103/physrevb.44.2589 9999827

[b22] Martínez-Lillo J. . Enhancing the magnetic coupling of oxalato-bridged Re^IV^_2_M^II^ (M=Mn, Co, Ni, and Cu) trinuclear complexes via peripheral halide ligand effects. Inorg. Chem. 50, 5731–5739 (2011). 21612191 10.1021/ic200531s

[b23] Martínez-Lillo J. . Ferromagnetic coupling and spin canting behaviour in heterobimetallic Re^IV^M^II/III^ (M=Co^II/III^, Ni^II^) species. Dalton Trans. 42, 1687–1695 (2013). 23154657 10.1039/c2dt31546g

[b24] González R. . Magnetic studies on hexaiodorhenate(IV) salts of univalent cations. spin canting and magnetic ordering in K_2_ReI_6_ with *T*_c_=24 K. Inorg. Chem. 42, 2512–2518 (2003). 12691556 10.1021/ic020531j

[b25] Martínez-Lillo J., Lloret F., Julve M. & Faus J. Spin canting in Re(IV) complexes: magnetic properties of [ReX_4_(bpym)] ( X=Cl and Br; bpym=2,2′-bipyrimidine). J. Coord. Chem. 62, 92–99 (2009).

[b26] Armentano D. & Martínez-Lillo J. Hexachlororhenate(IV) salts of ruthenium(III) cations: x-ray structure and magnetic properties. Inorg. Chim. Acta 380, 118–124 (2012).

[b27] Mousavi M., Béreau M. V., Duhayon C. & Sutter J.-P. K_2_Re(NCS)_6_: a weak ferromagnet. C. R. Chimie 15, 924–928 (2012).

[b28] Chiozzone R. . Synthesis, crystal structure, and magnetic properties of tetraphenylarsonium tetrachloro(oxalato)rhenate(IV) and bis(2,2′-bipyridine)tetrachloro(μ-oxalato)copper(II)rhenium(IV). Inorg. Chem. 38, 4745–4752 (1999). 11671200 10.1021/ic990376h

[b29] Cuevas A. . Rhenium(IV)−copper(II) heterobimetallic complexes with a bridge malonato ligand. synthesis, crystal structure, and magnetic properties. Inorg. Chem. 43, 7823–7831 (2004). 15554648 10.1021/ic0493853

[b30] Rouschias G. & Wilkinson W. The chemistry of rhenium–nitrile complexes. J. Chem. Soc. A 489–496 (1968).

[b31] Kunz P. C., Kurz P., Spingler B. & Alberto R. Attempted abstraction of the halogenides in (HNEt_3_)[Re(CH_3_CN)_2_Cl_4_] and crystal structures of cis-[Re(CH_3_CN)_2_Cl_4_]·CH_3_CN and cis-[Re(NHC(OCH_3_)CH_3_)_2_Cl_4_]. Z. Anorg. Allg. Chem. 633, 2753–2756 (2007).

[b32] Volkov S. V. . Synthesis and structure of rhenium(IV) chloride complexes with acetonitrile. Russ. J. Inorg. Chem. 52, 186–190 (2007).

[b33] Chiozzone R. . A novel series of rhenium-bipyrimidine complexes: synthesis, crystal structure and electrochemical properties. Dalton Trans. 653–660 (2007). 17268598 10.1039/b615136a

[b34] Giriat G., Wang W., Attfield J. P., Huxley A. D. & Kamenev K. V. Turnbuckle diamond anvil cell for high-pressure measurements in a superconducting quantum interference device magnetometer. Rev. Sci. Instrum. 81, 073905 (2010). 20687740 10.1063/1.3465311

[b35] Quintero P. A. . Pressure-induced enhancement of the magnetic anisotropy in Mn(N(CN)_2_)_2_. Phys. Rev. B 91, 014439 (2015).

[b36] Brinzari T. V. . Local lattice distortions in Mn[N(CN)_2_]_2_ under pressure. Inorg. Chem. 55, 1956–1961 (2016). 26863096 10.1021/acs.inorgchem.5b01870

[b37] Thirunavukkuarasu K. . Pressure dependence of the exchange anisotropy in an organic ferromagnet. Phys. Rev. B 91, 014412 (2015).

[b38] Tian D. . The metallic state in neutral radical conductors: dimensionality, pressure and multiple orbital effects. J. Am. Chem. Soc. 137, 14136–14148 (2015). 26513125 10.1021/jacs.5b08254

[b39] Coronado E. . Pressure-induced magnetic switching and linkage isomerism in K_0.4_Fe_4_[Cr(CN)_6_]_2.8_·16H_2_O: x-ray absorption and magnetic circular dichroism studies. J. Am. Chem. Soc. 130, 15519–15532 (2008). 18937456 10.1021/ja8047046

[b40] Kozlenko D. P. . Effect of high pressure on the crystal structure, magnetic, and vibrational properties of multiferroic RbFe(MoO_4_)_2_. Phys. Rev. B 87, 014112 (2013).

[b41] Shum W. W., Her J.-H., Stephens P. W., Lee Y. & Miller J. S. Observation of the pressure dependent reversible enhancement of T_c_ and loss of the anomalous constricted hysteresis for [Ru_2_(O_2_CMe)_4_]_3_[Cr(CN)_6_]. Adv. Mater. 19, 2910–2913 (2007).

[b42] McConnell A. C., Bell J. D. & Miller J. S. Pressure-induced transition from an antiferromagnet to a ferrimagnetic for Mn^II^(TCNE)[C_4_(CN)_8_]_1/2_ (TCNE=Tetracyanoethylene). Inorg. Chem. 51, 9978–9982 (2012). 22938759 10.1021/ic3014865

[b43] DaSilva J.-G. & Miller J. S. Pressure induced transition from spin glass-like behavior to a metamagnet exhibiting weak ferromagnetism observed for decamethylferrocenium hexacyanobutadiene, [FeCp*_2_]^·+^[HCBD]^·−^. Dalton Trans. 42, 8334–8338 (2013). 23612861 10.1039/c3dt50628b

[b44] Kumar Singh S. & Rajaraman G. Deciphering the origin of giant magnetic anisotropy and fast quantum tunnelling in Rhenium(IV) single-molecule magnets. Nat. Commun. 7, 10669 (2016). 26883278 10.1038/ncomms10669 PMC4757791

[b45] Moggach S. A., Allan D. R., Parsons S. & Warren J. E. Incorporation of a new design of backing seat and anvil in a Merrill-Bassett diamond anvil cell. J. Appl. Crystallogr. 41, 249–251 (2008).

[b46] Piermarini G. J., Block S., Barnett J. D. & Forman R. A. Calibration of the pressure dependence of the R1 ruby fluorescence line to 195 kbar. J. Appl. Phys. 46, 2774–2780 (1975).

[b47] Parsons S. ECLIPSE. The University of Edinburgh, Edinburgh, UK (2004).

[b48] Sheldrick G. M. SADABS. The University of Göttingen, Göttingen, Germany (1996).

[b49] Sheldrick G. M. SHELXT—integrated space-group and crystalstructure determination. Acta Cryst. A 71, 3–8 (2015). 10.1107/S2053273314026370 PMC4283466 25537383

[b50] Dolomanov O. V., Bourhis L. J., Gildea R. J., Howard J. A. K. & Puschmann H. OLEX2: a complete structure solution, refinement and analysis program. J. Appl. Cryst. 42, 339–341 (2009).

[b51] Thorn A., Dittrich B. & Sheldrick G. M. Enhanced rigid-bond restraints. Acta Cryst. A 68, 448–451 (2012).

[b52] Cliffe M. J. & Goodwin A. L. *PASCal*: a principal axis strain calculator for thermal expansion and compressibility determination. J. Appl. Cryst. 45, 1321–1329 (2012).

