# Supplementary material for: Pressure induced enhancement of the magnetic ordering temperature in rhenium(IV) monomers
Source: Nat Commun. 2016 Dec 21;7:13870. doi: 10.1038/ncomms13870 (PMC5187583; doi:10.1038/ncomms13870)
Supplement: Supplementary Information — Supplementary Figures, Supplementary Tables, Supplementary Notes, Supplementary Methods and Supplementary References. [file ncomms13870-s1.pdf]

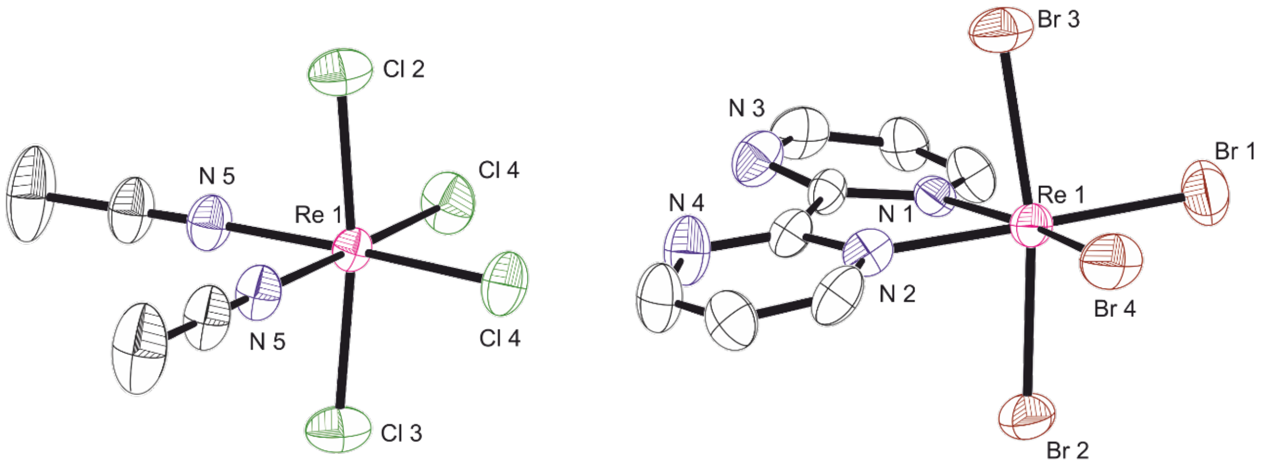

Supplementary Figure 1. **Crystal structures of 1 and 2.** ORTEP drawing of the mononuclear [$ReCl_4(MeCN)_2$] and [$ReBr_4$(bpym)] complexes of **1** (left) and **2** (right) at ambient pressure. The ellipsoids are depicted at 30 % occupancy. H atoms and solvent molecules of crystallization have been omitted for clarity.

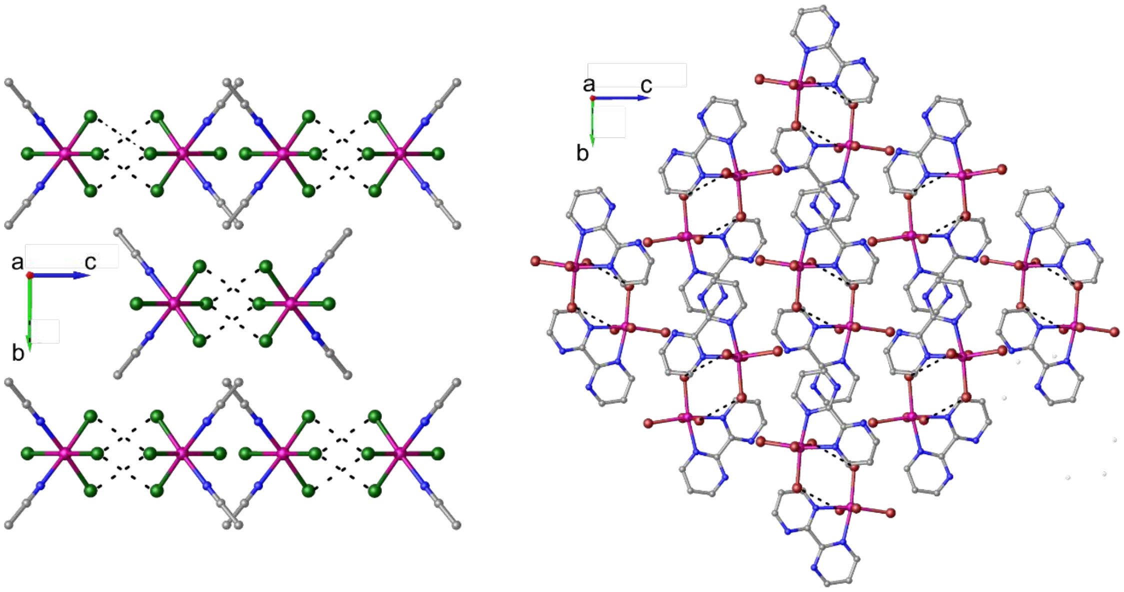

Supplementary Figure 2. Packing diagrams for 1 and 2. View along the crystallographic *a*-axis illustrating the layered and herringbone type structures of **1** (left) and **2** (right) in the crystal. Rhenium, chlorine, bromine, nitrogen, and carbon atoms are shown as pink, green, red, blue, and grey balls, respectively. H atoms and solvent molecules of crystallisation have been omitted for clarity.

a)

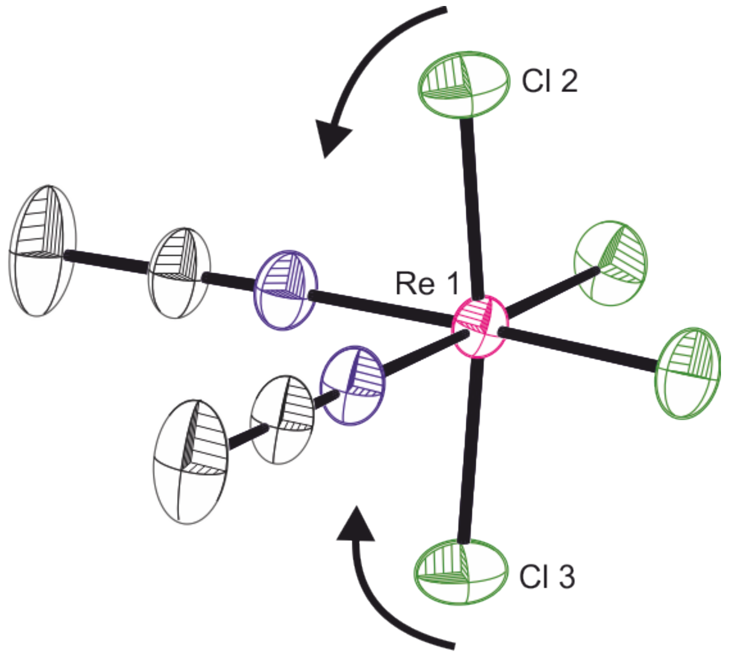

b)

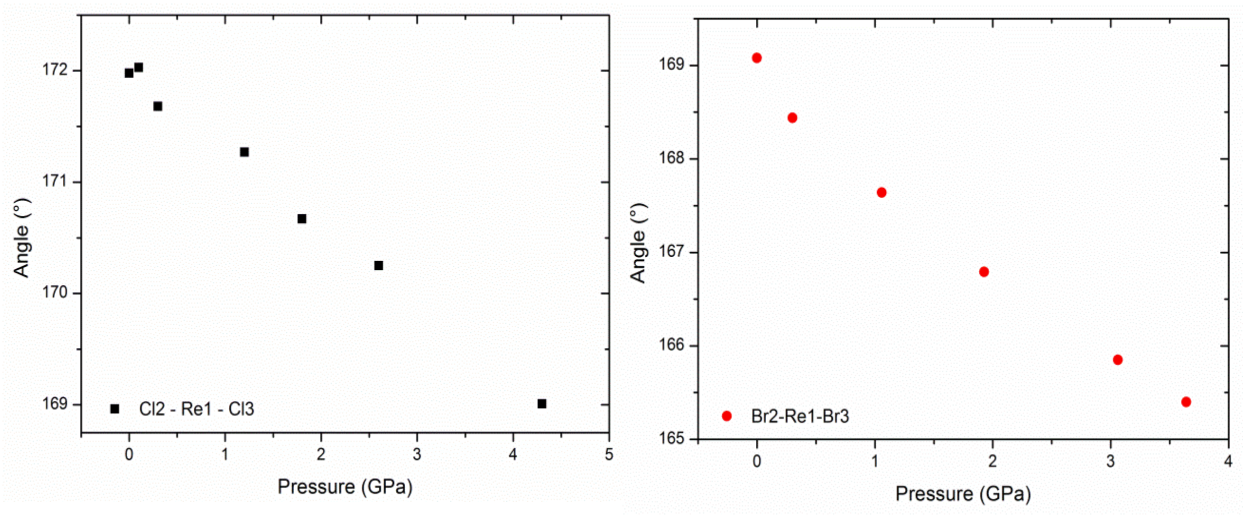

Supplementary Figure 3. Axial distortion of the Re^{IV} geometry with pressure. (**a**) View of the axial distortion of the Re^{IV} octahedral geometry with pressure in [$ReCl_4(MeCN)_2$] (**1**). (**b**) Variation of the halide-rhenium-halide angle [X-Re-X, X = Cl(**1**) and Br(**2**)] with pressure for **1** (left) and **2** (right).

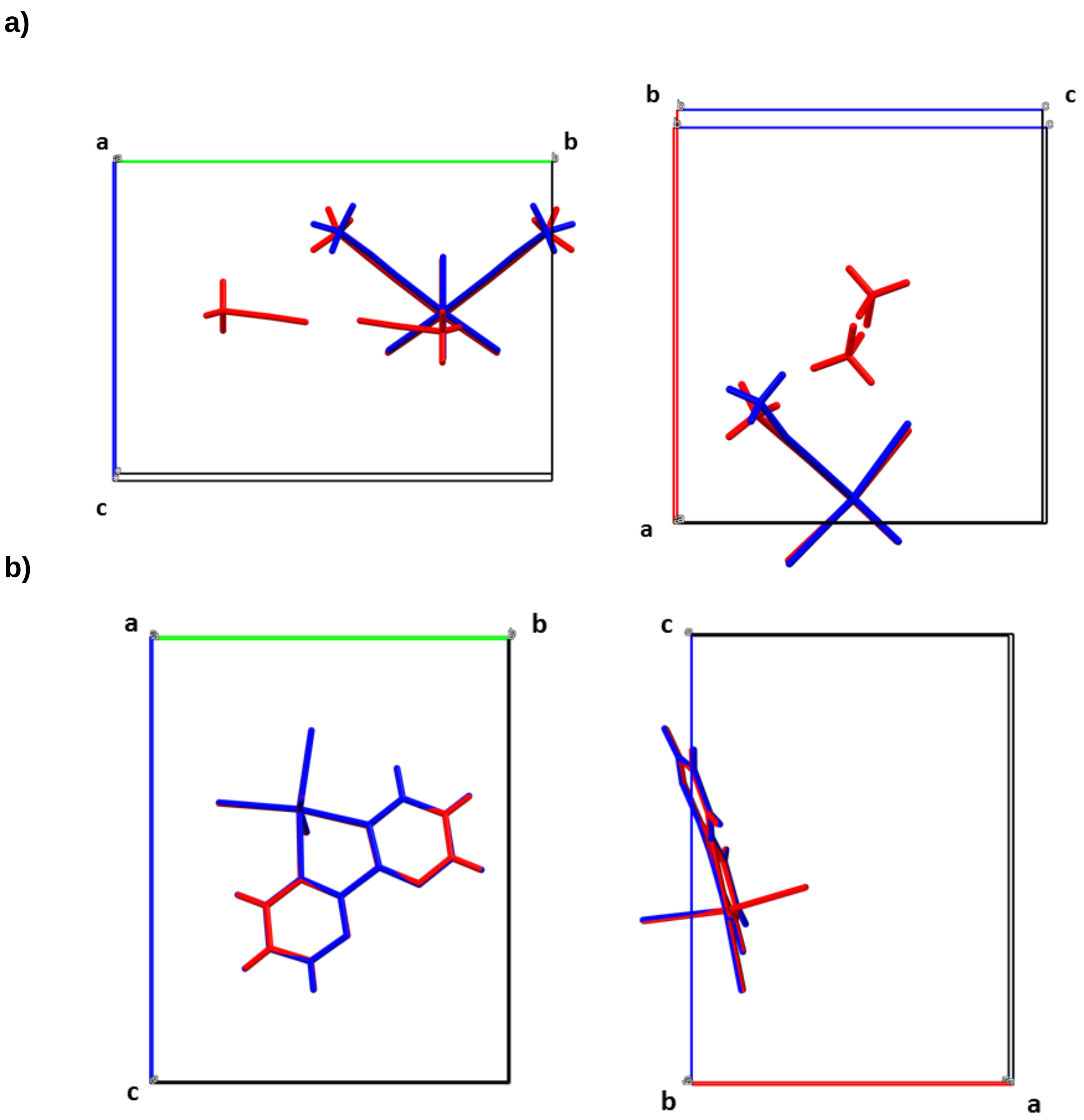

Supplementary Figure 4. Overlap of the *T* = 4 K and *T* = 300 K structures. (**a**) View of [$ReCl_4(MeCN)_2$]·MeCN (**1**) down the *a* (left) and *b* (right) axes with the high- and low-temperature structures overlapped. The blue structure is at *T* = 4 K and the red structure is at *T* = 300 K. (**b**) View of [$ReBr_4$(bpym)] (**2**) down the *a* (left) and *b* axes overlapped. Note the excellent overlap indicating minimal structure differences. Compound **1** undergoes a volume contraction of ~4.8 %, while compound **2** undergoes a contraction of ~3.2 %. The contraction in both compounds occurs across all axes but is more isotropic in compound **2**. In compound **1** there is a smaller contraction in the *b* axis.

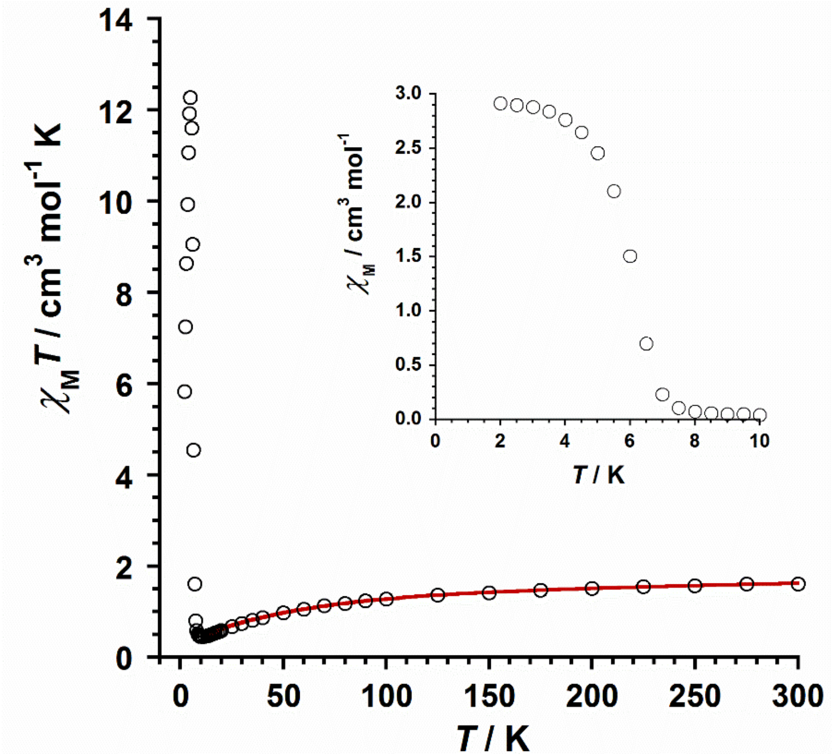

Supplementary Figure 5. Plot of $\chi_M T$ *versus* T obtained for compound 1 in the T = 2–300 K range. The solid red line represents the best-fit of the experimental data (o) in the temperature range 20–300 K, with $|D_{EXP}|$ = 37.1 cm^{-1}. The inset shows the temperature dependence of the magnetic susceptibility in the T = 2–10 K range.

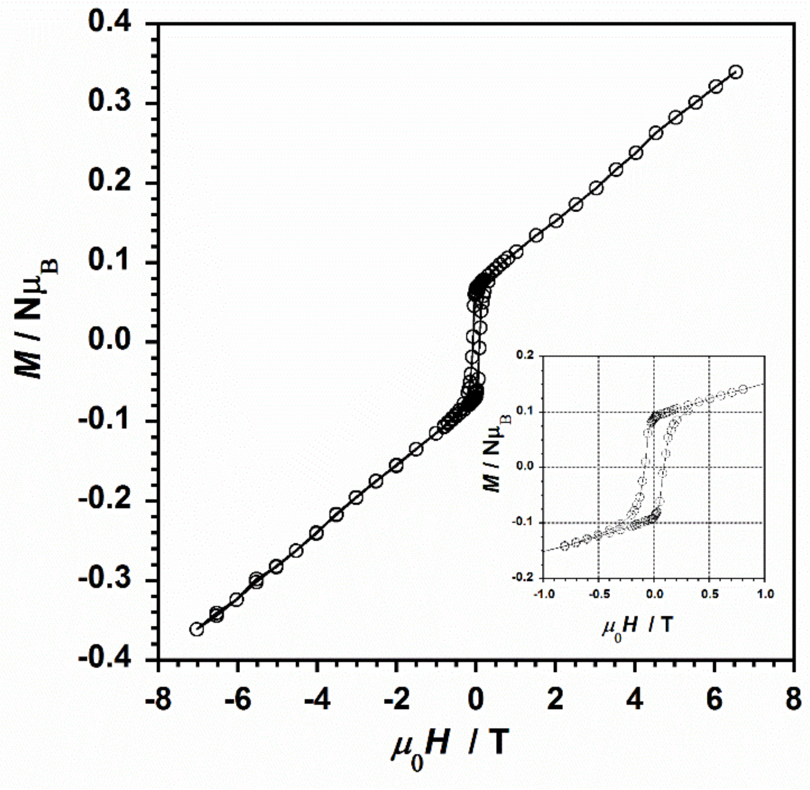

Supplementary Figure 6. Variable-field magnetisation data for 1 at T = 2.0 K. The inset shows the hysteresis loop of **1** at 2.0 K in the field range −1.0 to +1.0 T with H_c = 850 G and M_r = 0.068 μ_B.

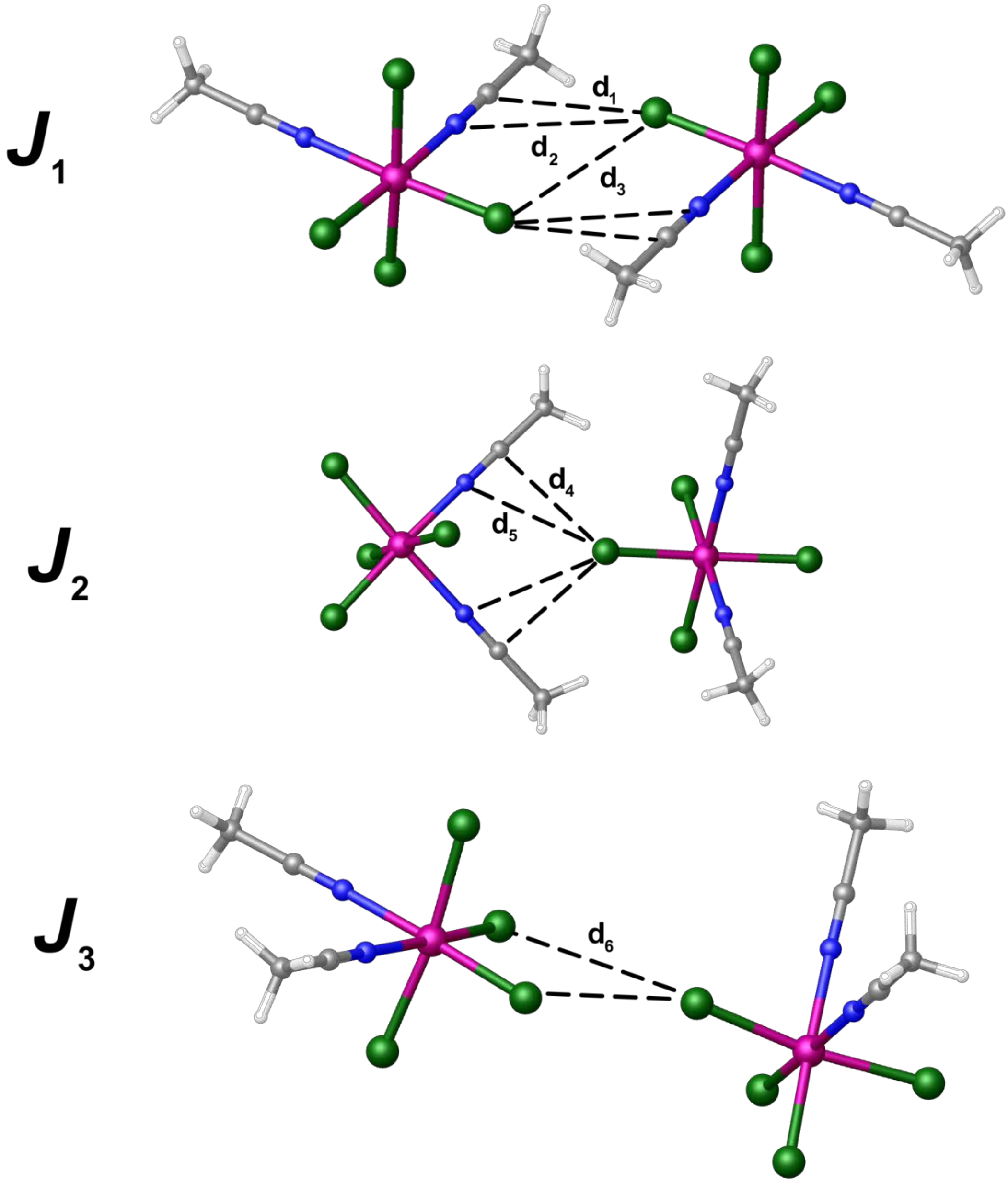

Supplementary Figure 7. Calculated intermolecular interactions for 1. The three intermolecular contacts which can transmit magnetic exchange (J_1-J_3) in **1**. The shortest contacts that are sensitive to pressure are displayed as dashed lines. Rhenium, chlorine, nitrogen, carbon and hydrogen atoms are shown in pink, green, blue, grey and white balls, respectively.

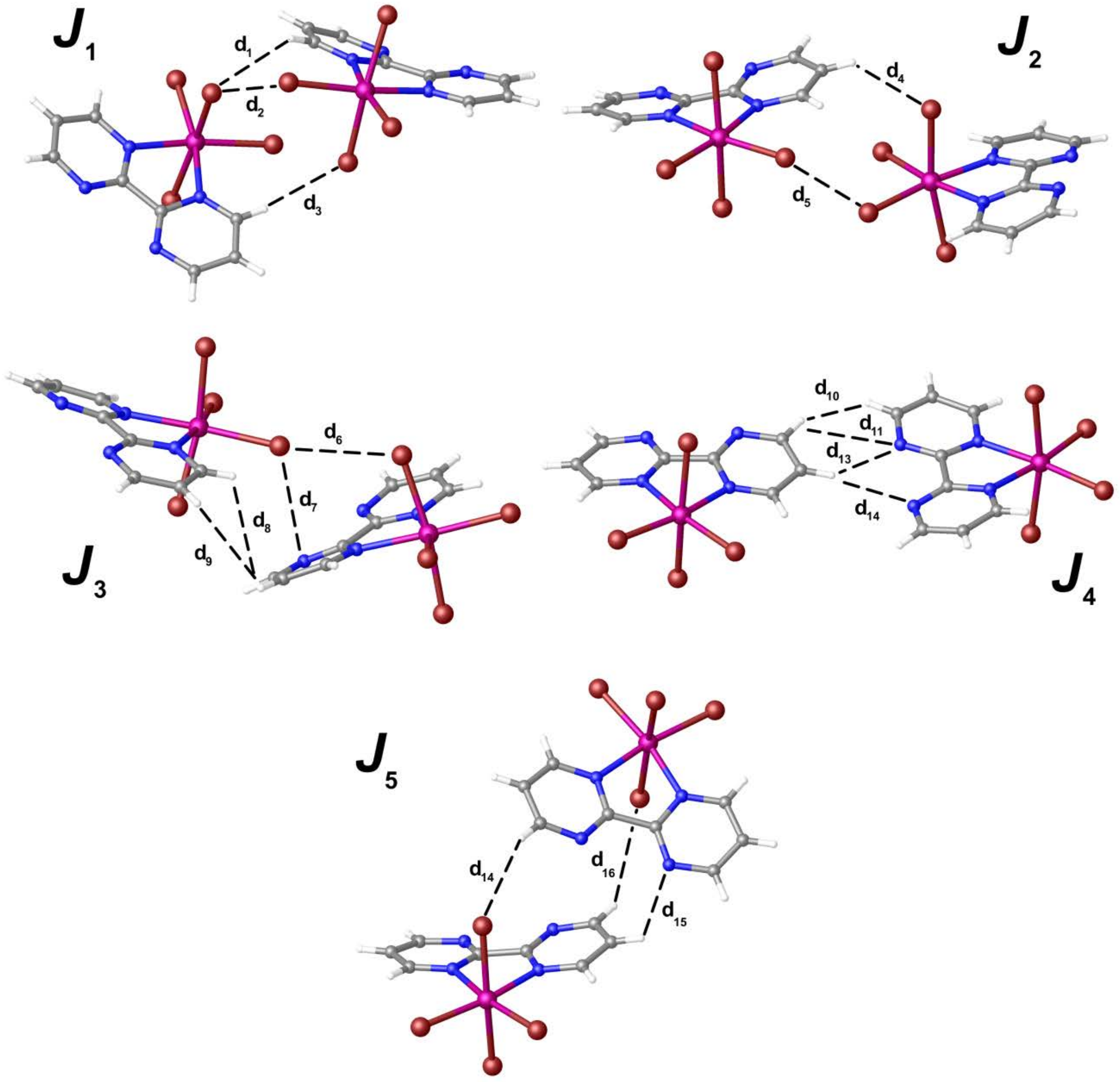

Supplementary Figure 8. Calculated intermolecular interactions for 2. The five intermolecular interactions which can transmit magnetic exchange (J_1-J_5) in **2**. The shortest contacts that are pressure sensitive are displayed as dashed lines. Rhenium, bromine, nitrogen, carbon and hydrogen atoms are shown in pink, red, blue, grey and white balls, respectively.

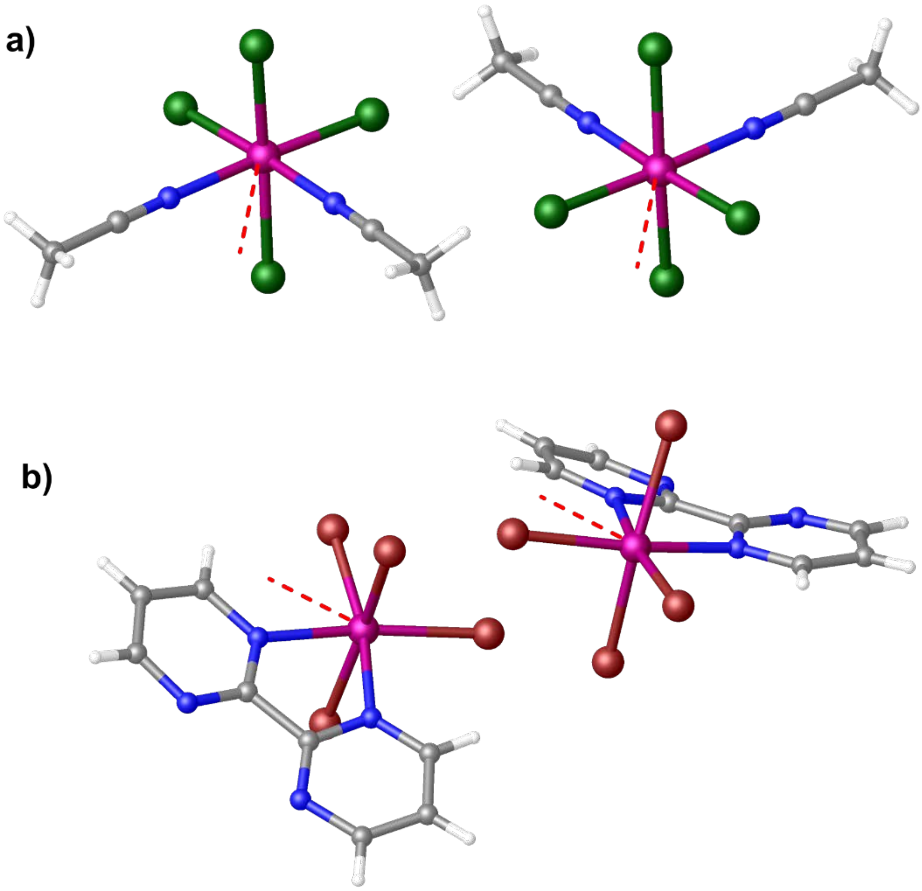

Supplementary Figure 9. View of the orientation of the *D* tensors in 1 and 2. Relative orientation of the *D* tensors on two neighbouring molecules in **1** (a) and **2** (b). In both cases, these orientations lead to contacts involving the J_1 constant at ambient pressure. The red dashed lines represent the *z* axis of the *D* tensor.

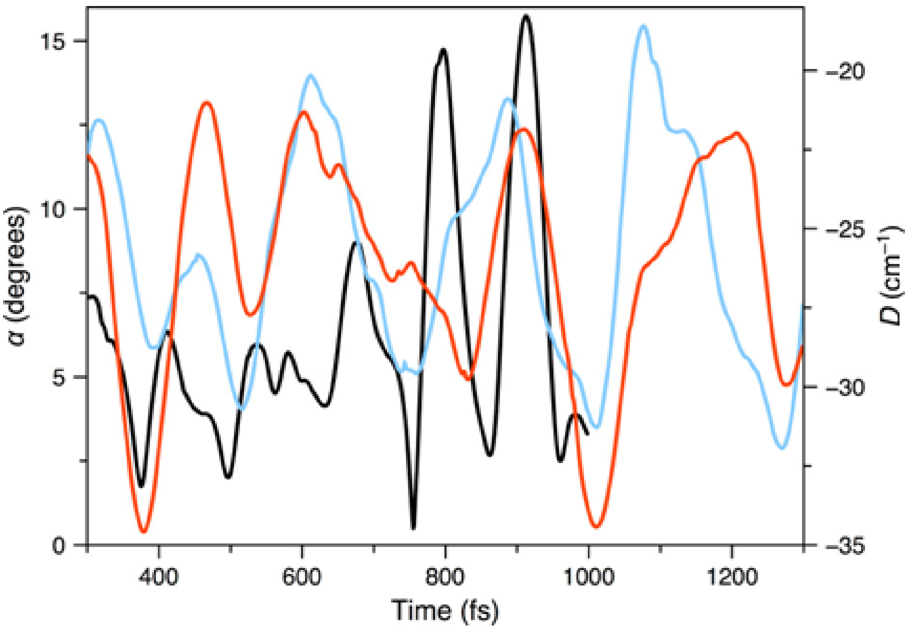

Supplementary Figure 10. Molecular dynamics on 1. Time evolution at 5 K of the α_{SIM} angle (black line) and D_{SIM} parameter of the two [$ReCl_4(MeCN)_2$] unities (blue and red lines) involved in the J_1 magnetic exchange.

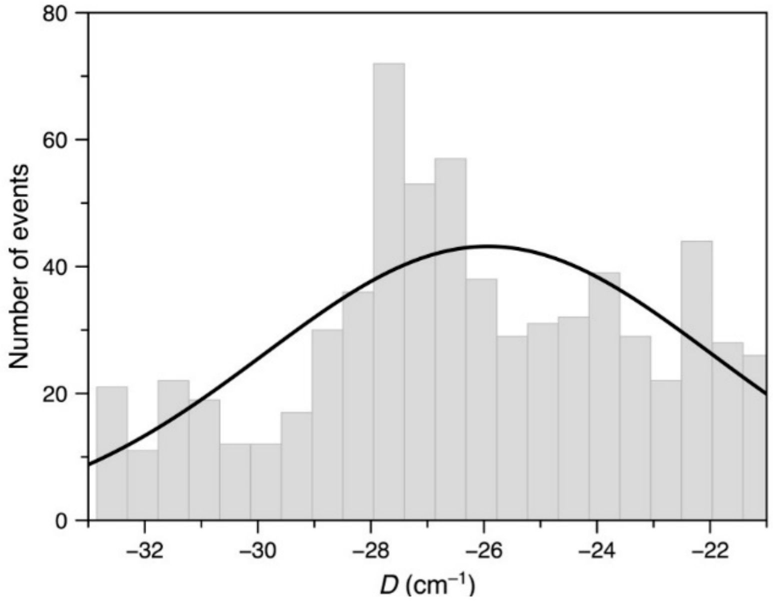

Supplementary Figure 11. **Histogram of the average axial *zfs* parameter value in 1**. Distribution of the average D_{SIM} value of the $[ReCl_4(MeCN)_2]$ units, involved in the J_1 magnetic exchange, obtained from molecular dynamics performed at 5 K. The bars show the number of events for each interval value and the solid black line is the best-fit to a Gaussian curve.

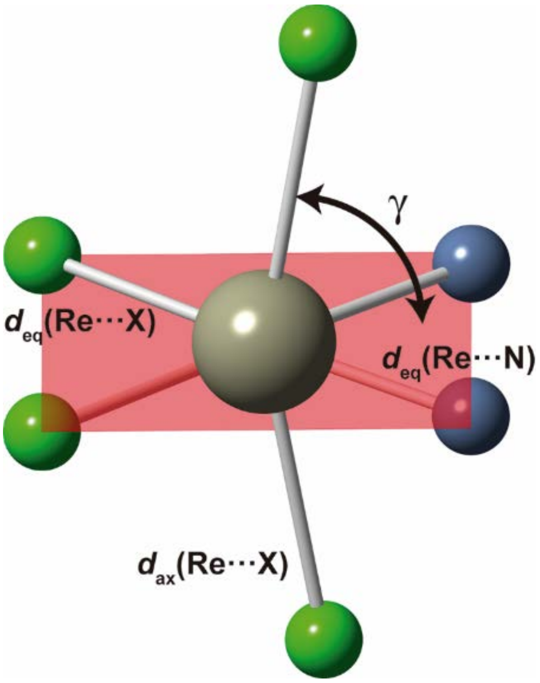

Supplementary Figure 12. Model illustrating the angles and distances affected by pressure. The relevant geometrical parameters in **1** and **2** [X = Cl(**1**), Br(**2**)] affected by applied external pressure.

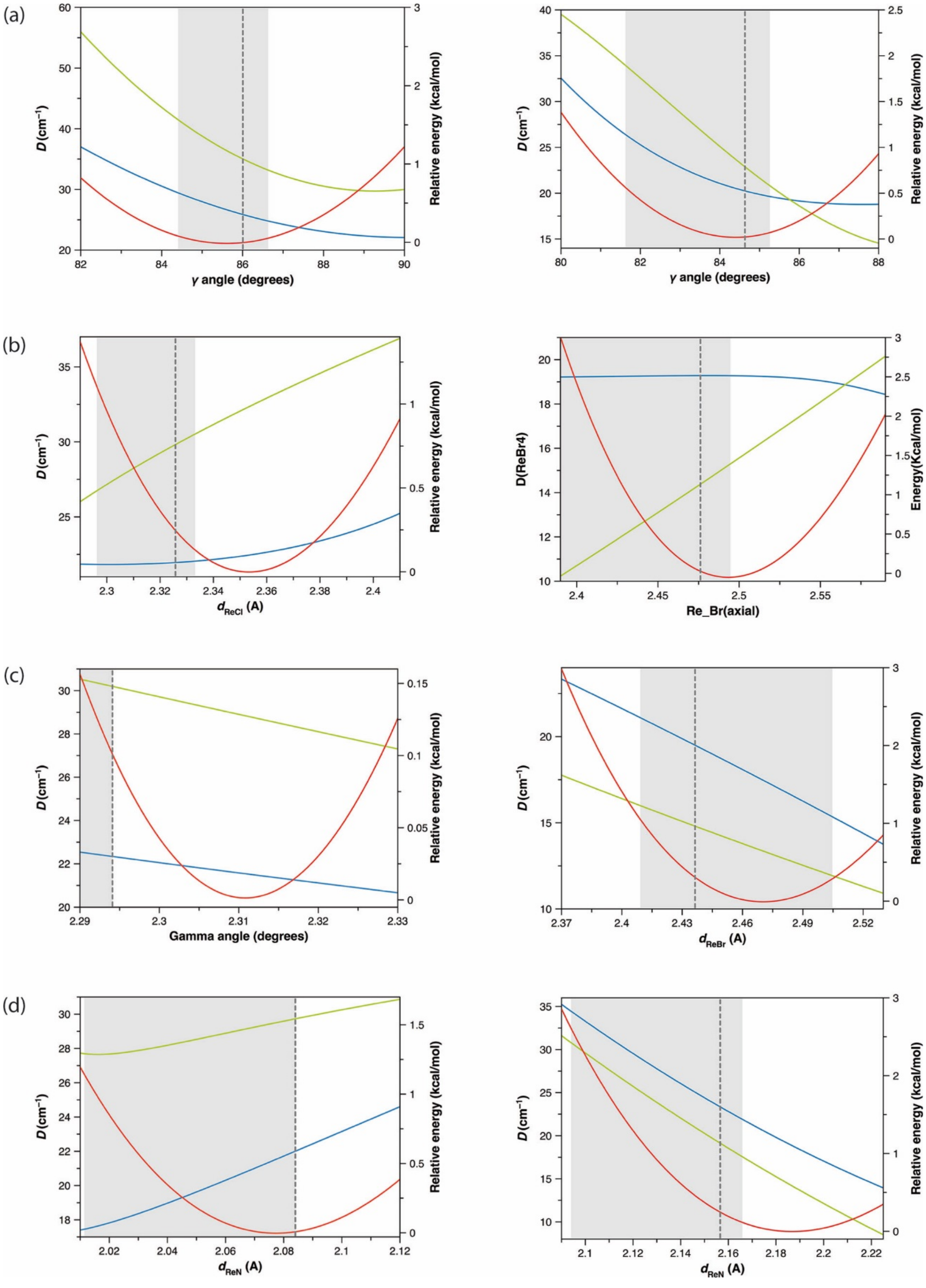

Supplementary Figure 13. Influence of several geometrical parameters on the axial anisotropy parameter (|*D*|, in cm^{-1}) for 1 (left) and 2 (right). Absolute values of *D* are provided since the large [and overestimated] calculated *E*/*D* values that are close to 1/3 can lead to a change in the sign of *D*.

The grey stripe delimits the regions where the experimental values are found, and the dashed line shows the average value for the studied geometrical parameter in the experimental ambient structure. Blue and green lines are the results obtained from CAS and PBE-DFT methods. CAS [rather than NEVPT2] results were used due to problems associated with reaching convergence criterion for some of the studied molecular geometries in the latter. The red line shows the evolution of the electronic energy (in kcal/mol) with the studied geometrical parameter.

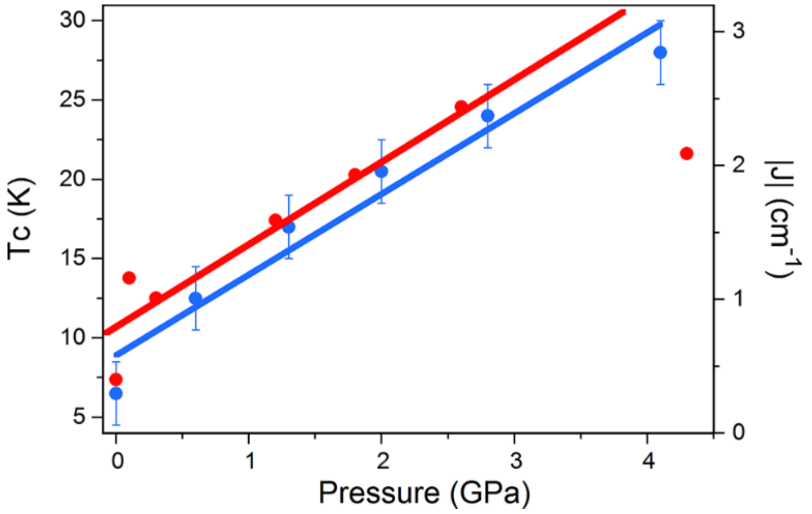

Supplementary Figure 14. Variation with pressure of the T_c and J values in 1. Pressure dependence of the ordering temperature, T_c (blue circles), and the strongest magnetic coupling, J (red circles), in **1**.

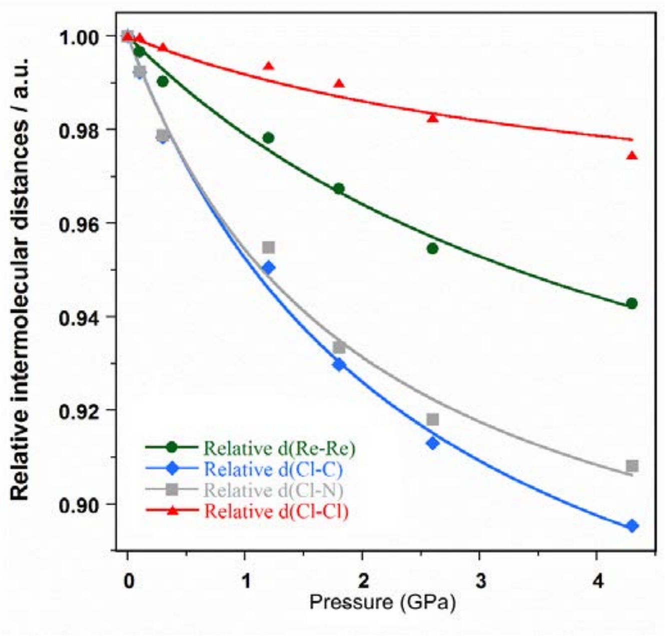

Supplementary Figure 15. Relative intermolecular distances (d) between adjacent [$ReCl_4(MeCN)_2$] complexes in 1. Variation with pressure of the intermolecular Re···Re, Cl···C, Cl···N and Cl···Cl distances involved in the contacts mediating the J_1 magnetic exchange between two adjacent [$ReCl_4(MeCN)_2$] complexes in **1**.

Supplementary Table 1. Crystallographic details for 1. For all structures: $C_6H_9Cl_4N_3Re$, M_r = 451.17, orthorhombic, *Pnma*, Z = 4. Experiments were performed at 300 K. Absorption was corrected by multi-scan methods, *SADABS* (Siemens, 1996). H atom parameters were not refined.

	**ambient**	**0.1 GPa**	**0.3 GPa**	**1.2 GPa**
Crystal data				
REFCODE	966619	966620	966621	966622
a, b, c (Å)	10.4093(4)<br>13.5203(5)<br>9.8134(3)	10.3829(6)<br>13.4805(9)<br>9.7632(11)	10.2971(5)<br>13.4016(9)<br>9.6238(10)	10.1180(6)<br>13.259(2)<br>9.3321(6)
V (Å^3)	1381.11(8)	1366.5(2)	1328.06(18)	1251.9(2)
Radiation type	Mo $K\alpha$	Synchrotron - Diamond Light Source Beamline I19, λ = 0.48590 Å	Synchrotron - Diamond Light Source Beamline I19, λ = 0.48590 Å	Synchrotron - Diamond Light Source Beamline I19, λ = 0.48590 Å
μ (mm^{-1})	9.54	3.53	3.63	3.85
Crystal size (mm)	0.12 × 0.09 × 0.06	0.08 × 0.07 × 0.05	0.08 × 0.07 × 0.05	0.09 × 0.07 × 0.06
Data collection				
T_{min}, T_{max}	0.530, 0.746	0.51, 0.62	0.51, 0.61	0.546, 0.744
No. of measured, independent and observed [$I > 2.0\sigma(I)$] reflections	8215, 1779, 1604	11534, 1512, 1357	11196, 1470, 1315	7735, 798, 736
R_{int}	0.030	0.046	0.044	0.040
θ_{max} (°)	28.272	20.292	20.334	17.637
Refinement				
$R[F^2 > 2\sigma(F^2)]$, $wR(F^2)$, S	0.019, 0.019, 1.07	0.030, 0.028, 0.91	0.026, 0.022, 0.91	0.018, 0.020, 0.88
No. of reflections	1604	1357	1315	736
No. of parameters	66	66	66	66
No. of restraints	38	38	38	38
$\Delta\rangle_{max}$, $\Delta\rangle_{min}$ (e Å^{-3})	0.63, -0.81	1.36, -2.46	0.84, -1.53	0.55, -0.53

	**1.8 GPa**	**2.6 GPa**	**4.3 GPa**
Crystal data			
REFCODE	966623	966624	966625
a, *b*, *c* (Å)	10.0281(5)<br>13.122(2)<br>9.1585(5)	9.9548(10)<br>12.959(5)<br>9.0069(11)	9.835(2)<br>12.816(15)<br>8.746 (3)
V (Å^3)	1205.1(2)	1161.9(5)	1102.4(13)
Radiation type	Synchrotron - Diamond Light Source Beamline I19, λ = 0.48590 Å	Synchrotron - Diamond Light Source Beamline I19, λ = 0.48590 Å	Synchrotron - Diamond Light Source Beamline I19, λ = 0.48590 Å
μ (mm^{-1})	4.00	4.15	4.37
Crystal size (mm)	0.09 × 0.07 × 0.06	0.09 × 0.07 × 0.06	0.09 × 0.07 × 0.06
Data collection			
T_{min}, T_{max}	0.560, 0.744	0.439, 0.744	0.11, 0.49
No. of measured, independent and observed [$I > 2.0\sigma(I)$] reflections	8162, 917, 808	5495, 537, 475	1894, 527, 400
R_{int}	0.038	0.061	0.068
θ_{max} (°)	18.9	15.7	17.7
Refinement			
$R[F^2 > 2\sigma(F^2)]$, $wR(F^2)$, S	0.023, 0.023, 1.00	0.029, 0.033, 1.01	0.070, 0.051, 1.11
No. of reflections	808	475	400
No. of parameters	66	66	51
No. of restraints	38	38	26
$\Delta\rangle_{max}$, $\Delta\rangle_{min}$ (e Å^{-3})	0.70, -0.56	0.67, -0.58	1.51, -1.50

Supplementary Table 2. Crystallographic details for 2. For all structures: $C_8H_6Br_4N_4Re$, M_r = 664.01, orthorhombic, $P2_12_12_1$, Z = 4 with the exception of data collected at 3.06 GPa and 3.64 GPa which have undergone a transformation to a new phase, monoclinic, $P2_1$, Z = 4. Experiments were performed at 298 K. Absorption was corrected by multi-scan methods, *SADABS* (Siemens, 1996). H atom parameters were not refined.

	**ambient**	**0.30 GPa**	**1.06 GPa**	**1.59 GPa**
Crystal data				
REFCODE	1453406	1453399	1453400	1453401
Space Group	$P2_12_12_1$	$P2_12_12_1$	$P2_12_12_1$	$P2_12_12_1$
a, b, c (Å)	9.6089(3)<br>10.8491(3)<br>13.4542(4)	9.4226(3)<br>10.7168(3)<br>13.2648(4)	9.2129(5)<br>10.5779(6)<br>13.0532(34)	9.1029(10)<br>10.5419(9)<br>12.9562(8)
V (Å^3)	1402.5(1)	1339.48(7)	1272.0	1243.30(19)
Radiation type	Mo $K\alpha$	Mo $K\alpha$	Mo $K\alpha$	Mo $K\alpha$
μ (mm^{-1})	20.042	20.985	22.097	22.609
Crystal size (mm)	0.08 x 0.05 x 0.01	0.08 x 0.05 x 0.01	0.08 x 0.05 x 0.01	0.08 x 0.05 x 0.01
Data collection				
T_{min}, T_{max}	0.4676, 0.7456	0.7037,1.0000	0.7066,1.000	0.5463, 1.000
No. of measured, independent and observed [$I > 2.0\sigma(I)$] reflections	22540,3265,2694	7781, 1800, 1710	7464,1707, 1633	5207,1273, 1170
R_{int}	0.0349	0.0288	0.0284	0.0654
θ_{max} (°)	27.89	27.07	26.79	25.59
Refinement				
$R[F^2 > 2\sigma(F^2)]$, $wR(F^2)$, S	0.0307,0.0514, 1.037	0.0300,0.0760, 1.212	0.0284, 0.0638, 1.104	0.0525, 0.1326, 1.189
No. of reflections	2694	1710	1633	1273
No. of parameters	154	148	130	70
No. of restraints	0	0	0	0
$\Delta\rangle_{max}$, $\Delta\rangle_{min}$ (e Å^{-3})	1.721, -1.312	1.074, -0.980	1.057, -0.837	2.690, -1.736

	**1.93 GPa**	**3.06 GPa**	**3.64 GPa**	**Ambient in cell**
Crystal data				
REFCODE	1453402	1453403	1453404	1453405
Space group	$P2_12_12_1$	$P2_1$	$P2_1$	$P2_12_12_1$
a, b, c (Å)<br>β (°)	9.0638(3)<br>10.4978(3)<br>12.9039(4)	8.9935(11)<br>12.7551(14)<br>10.4880(11)<br>91.208(4)	8.8730(10)<br>12.7780(9)<br>10.3459(10)<br>92.250(9)	9.6184(4)<br>10.8488(4)<br>13.4471(4)
V (Å^3)	1227.8(1)	1186.20(10)	1186.20(10)	1403.18(9)
Radiation type	Mo $K\alpha$	Mo $K\alpha$	Mo $K\alpha$	Mo $K\alpha$
μ (mm^{-1})	22.894	23.697	23.697	19.988
Crystal size (mm)	0.08 x 0.05 x 0.01	0.08 x 0.05 x 0.01	0.08 x 0.05 x 0.01	0.08 x 0.05 x 0.01
Data collection				
T_{min}, T_{max}	0.6753,1.0000	0.6440, 1.000	0.4713, 1.000	0.4549,0.7454
No. of measured, independent and observed [$I > 2.0\sigma(I)$] reflections	7145,1647, 1586	6556, 1919, 1809	4857, 1793, 1711	920, 920, 830
R_{int}	0.0276	0.0320	0.0309	Merged data
θ_{max} (°)	26.87	26.976	26.976	26.981
Refinement				
$R[F^2 > 2\sigma(F^2)]$, $wR(F^2)$, S	0.0281, 0.0667, 1.189	0.0335, 0.0739, 1.098	0.0335, 0.0739, 1.097	0.0457, 0.1126, 1.210
No. of reflections	1647	1919	1793	920
No. of parameters	94	139	139	70
No. of restraints	0	1	1	0
$\Delta\rangle_{max}$, $\Delta\rangle_{min}$ (e Å^{-3})	0.900, -1.025	1.098, -1.026	1.226, -0.7663	1.721, -1.312

Supplementary Table 3. Void volume changes with pressure in 1. Table containing the % volume and the void volume for each studied pressure in compound **1**; probe radius 1.2 Å and grid spacing 0.7 Å.

**Pressure (GPa)**	**% volume**	**Void volume (Å^3)**
Ambient	29.8	411.88
0.10	29.3	400.20
0.30	28.3	376.28
1.20	26.7	334.48
1.80	26.0	313.52
2.60	24.8	288.21
4.30	21.6	237.74

Supplementary Table 4. Shortest intermolecular Re-Cl···Cl-Re distances in 1. Table containing selected intermolecular Re-Cl···Cl-Re distances as function of pressure in **1**.

**Pressure (GPa)**	**Distance (Å)**	
	**Re-Cl(2)…Cl(4)-Re**	**Re-Cl(4)…Cl(4)'-Re**
Ambient	3.922(1)	4.014(2)
0.10	3.877(2)	4.015(3)
0.30	3.795(2)	4.005(2)
1.20	3.623(2)	3.989(4)
1.80	3.535(2)	3.974(4)
2.60	3.465(3)	3.945(7)
4.30	3.362(11)	3.91(2)

Supplementary Table 5. Shortest intermolecular Re-Br···Br-Re distances in 2. Table containing the shortest intermolecular Re-Br···Br-Re distances as function of pressure in **2**. At 3.06 GPa **2** exhibits a structural transition to a new monoclinic $P2_1$ form, and two crystallographically independent $[ReBr_4(bpym)]$ molecules occur in the asymmetric unit. The data for both are included.

**Pressure (GPa)**	**Distance (Å)**		
	**Re-Br(1)···Br(4)'-Re (in $P2_12_12_1$)**<br><br>**Re-Br(1)···Br(8)-Re or Re-Br(5)···Br(4)-Re (in $P2_1$)**	**Re-Br(2)···Br(1)'-Re (in $P2_12_12_1$)**<br><br>**Re-Br(2)···Br(5)-Re or Re-Br(1)···Br(6)-Re (in $P2_1$)**	**Re-Br(2)···Br(4)'-Re (in $P2_12_12_1$)**<br><br>**Re-Br(6)···Br(8)-Re or Re-Br(2)···Br(4)-Re (in $P2_1$)**
Ambient	3.9227(16)	3.8524(15)	3.8923(14)
0.30	3.835(3)	3.791(3)	3.820(3)
1.06	3.741(3)	3.725(3)	3.723(3)
1.59	3.694(6)	3.708(6)	3.680(5)
1.93	3.678(3)	3.686(3)	3.655(3)
3.06	3.610(8)	3.668(5)	3.633(8)
	3.623(4)	3.633(8)	3.590(5)
3.64	3.5652(4)	3.6067(3)	3.5780(2)
	3.6040(4)	3.6649(2)	3.5718(2)

Supplementary Table 6. Selected intramolecular angles and bond lengths for 1. Table containing intramolecular angles and Re-ligand bond lengths as function of pressure in **1**.

Pressure (GPa)	Distance (Å)				Angle (°)
	Re(1)-Cl(2)	Re(1)-Cl(3)	Re(1)-Cl(4)	Re(1)-N(5)	Cl(2)-Re(1)-Cl(3)
Ambient	2.3188(11)	2.3312(11)	2.2987(7)	2.085(2)	171.98(5)
0.10	2.3239(17)	2.3326(17)	2.2984(10)	2.085(3)	172.03(7)
0.30	2.3194(14)	2.3315(15)	2.2963(9)	2.088(3)	171.68(6)
1.20	2.3137(12)	2.3234(12)	2.2990(16)	2.082(5)	171.27(5)
1.80	2.3083(16)	2.3193(16)	2.2902(19)	2.082(7)	170.67(7)
2.60	2.302(3)	2.319(3)	2.287(3)	2.066(10)	170.25(11)
4.30	2.296(7)	2.306(7)	2.290(11)	2.01(3)	169.0(3)

Supplementary Table 7. Selected intramolecular angles and bond lengths for 2. Table containing intramolecular angles and bond lengths as function of pressure in **2**. At 3.06 GPa **2** exhibits a structural transition, and two crystallographically independent [$ReBr_4$(bpym)] molecules occur in the asymmetric unit. The data for both are included.

**Pressure (GPa)**	**Distance (Å)**						**Angle (°)**
	**Re(1)-Br(1) (in $P2_12_12_1$)**<br>**Re(1)-Br(1) or Re(2)-Br(5) (in $P2_1$)**	**Re(1)-Br(2) (in $P2_12_12_1$)**<br>**Re(1)-Br(2) or Re(2)Br(6) (in $P2_1$)**	**Re(1)-Br(3) (in $P2_12_12_1$)**<br>**Re(1)-Br(3) or Re(2)-Br(7) (in $P2_1$)**	**Re(1)-Br(4) (in $P2_12_12_1$)**<br>**Re(1)-Br(4) or Re(2)-Br(8) (in $P2_1$)**	**Re(1)-N(1) (in $P2_12_12_1$)**<br>**Re(1)-N(1) or Re(2)-N(5) (in $P2_1$)**	**Re(1)-N(2) (in $P2_12_12_1$)**<br>**Re(1)-N(2) or Re(2)-N(6) (in $P2_1$)**	**Br(2)-Re(1)-Br(3) (in $P2_12_12_1$)**<br>**Br(2)-Re(1)-Br(3) or Br(6)-Re(2)-Br(8) (in $P2_1$)**
Ambient	2.4352(10)	2.4567(10)	2.4915(10)	2.4381(10)	2.164(58)	2.147(69)	169.08(3)
0.30	2.4364(18)	2.453(2)	2.492(2)	2.433(2)	2.152(13)	2.125(14)	168.43(7)
1.06	2.433(17)	2.448(2)	2.492(2)	2.432(2)	2.111(13)	2.129(12)	167.59(7)
1.59	2.433(4)	2.427(5)	2.476(4)	2.433(4)	2.116(14)	2.142(13)	167.00(13)
1.93	2.4344(17)	2.442(2)	2.485(2)	2.426(2)	2.101(13)	2.116(12)	166.76(7)
3.06	2.427(4)<br>2.436(5)	2.427(6)<br>2.438(5)	2.478(5)<br>2.416(4)	2.428(5)<br>2.464(6)	2.099(13)<br>2.078(12)	2.110(14)<br>2.051(15)	166.22(13)<br>165.56(16)
3.64	2.430(4)<br>2.436(5)	2.431(5)<br>2.438(4)	2.476(3)<br>2.464(5)	2.424(3)<br>2.416(3)	2.090(12)<br>2.066(12)	2.090(16)<br>2.067(16)	166.09(12)<br>164.76(14)

Supplementary Table 8. Crystallographic details for 1 and 2 at *T* = 4 K. Structural parameters obtained for complexes **1** and **2** from single crystal X-ray diffraction at a temperature of 4 K.

	**Compound 1 @ 4 K**	**Compound 2 @ 4 K**
Crystal data		
REFCODE	1491360	1491361
Space Group	*Pnma*	$P2_12_12_1$
a, *b*, *c* (Å)	10.2610(2)<br>13.4766(3)<br>9.5069(2)	9.4883(3)<br>10.7299(4)<br>13.3288(5)
V (Å^3)	1314.65(5)	1356.99(8)
Radiation type	Mo$K\alpha$	Mo$K\alpha$
μ (mm^{-1})	10.024	20.715
Crystal size (mm)	0.18 x 0.15 x 0.11	0.20 x 0.16 x 0.14
Data collection		
T_{min}, T_{max}	0.4015, 0.7456	0.4680, 0.7456
No. of measured, independent and observed [$I > 2.0\sigma(I)$] reflections	15800, 1607, 1597	17483, 3162, 3104
R_{int}	0.0199	0.0251
θ_{max} (°)	27.849	27.957
Refinement		
$R[F^2 > 2\sigma(F^2)]$, $wR(F^2)$, S	0.0151, 0.0339, 1.265	0.0215, 0.0433, 1.049
No. of reflections	1607	3162
No. of parameters	88	154
No. of restraints	57	162
$\Delta\rangle_{max}$, $\Delta\rangle_{min}$ (e Å^{-3})	1.396, -1.022	1.335, -1.018

Supplementary Table 9. Intermolecular magnetic exchange values in 1 and 2. Calculated magnetic exchange constants (J_i / cm^{-1}) together with the intermolecular distances (d_i / Å) at ambient pressure and at high pressure.

	**Ambient Pressure**		**High Pressure**	
	J_i	d_i	J_i	d_i
	**Compound 1**			
J_1	– 0.40	d_1 = 3.46<br>d_2 = 3.83<br>d_3 = 4.01	– 2.09	d_1 = 3.10<br>d_2 = 3.48<br>d_3 = 3.91
J_2	– 0.18	d_4 = 3.24<br>d_5 = 3.45	– 3.71	d_4 = 2.89<br>d_5 = 3.06
J_3	– 0.05	d_6 = 3.92	– 0.74	d_6 = 3.36
	**Compound 2**			
J_1	– 1.91	d_1 = 2.92<br>d_2 = 3.85<br>d_3 = 3.28	– 5.52	d_1 = 2.62<br>d_2 = 3.65<br>d_3 = 2.77
J_2	– 1.00	d_4 = 3.01<br>d_5 = 3.92	– 2.55	d_4 = 2.71<br>d_5 = 3.59
J_3	+ 0.38	d_6 = 3.89<br>d_7 = 3.57<br>d_8 = 3.39<br>d_9 = 3.94	– 0.65	d_6 = 3.60<br>d_7 = 3.27<br>d_8 = 2.81<br>d_9 = 3.28
J_4	– 0.0004	d_{10} = 2.61<br>d_{11} = 3.11<br>d_{12} = 2.83<br>d_{13} = 2.67	– 0.0007	d_{10} = 2.30<br>d_{11} = 2.75<br>d_{12} = 2.43<br>d_{13} = 2.36

$\boldsymbol{J_5}$	– 0.14	d_{14} = 2.96<br>d_{15} = 3.07<br>d_{16} = 3.34	– 0.27	d_{14} = 2.67<br>d_{15} = 2.68<br>d_{16} = 3.30

Supplementary Table 10. Intermolecular magnetic exchange values in 1 and 2. Magnetic exchange constants (J_i / cm^{-1}) calculated in the gas phase and with a solvent model at ambient pressure and at high pressure.

	**Ambient Pressure**		**High Pressure**	
	**J_i / gas**	**J_i / solvent**	**J_i / gas**	**J_i / solvent**
	**Compound 1**			
J_1	– 0.27	– 0.40	– 1.79	– 2.09
J_2	– 0.21	– 0.18	– 3.94	– 3.71
J_3	– 0.25	– 0.05	– 2.44	– 0.74
	**Compound 2**			
J_1	– 2.25	– 1.91	– 6.60	– 5.52
J_2	– 1.21	– 1.00	– 3.24	– 2.55
J_3	+ 0.41	+ 0.38	– 0.69	– 0.65
J_4	– 0.0004	– 0.0004	– 0.0007	– 0.0007
J_5	– 0.16	– 0.14	– 0.33	– 0.27

Supplementary Table 11. **Comparison of the *g*, *D* and *E*/*D* parameters for 1 and 2**. *g*, *D* and *E*/*D* parameters for complexes **1** and **2** calculated through NEVPT2 and CAS methods at ambient pressure and at 4.3 GPa.

	**0.0 GPa**		**4.3 GPa**	
**Compound 1**	**NEVPT2**	**CAS**	**NEVPT2**	**CAS**
g_1	1.763	1.781	1.775	1.799
g_2	1.765	1.783	1.777	1.802
g_3	1.774	1.791	1.783	1.805
g_{ISO}	1.767	1.785	1.778	1.802
D / cm^{-1}	-30.00	-25.80	-22.70	-18.40
E / D	0.218	0.276	0.226	0.282
D_{SS}	-0.01	-0.01	-0.01	-0.01
D_{SO}	-30.00	-25.80	-22.70	-18.40
D_Q	-5.20	-5.80	-5.80	-6.10
D_D	-24.80	-20.30	-16.70	-12.30
	**0.0 GPa**		**3.6 GPa**	
**Compound 2**	**NEVPT2**	**CAS**	**NEVPT2**	**CAS**
g_1	1.765	1.790	1.765	1.791
g_2	1.767	1.787	1.771	1.793
g_3	1.780	1.796	1.790	1.813
g_{ISO}	1.767	1.785	1.778	1.802
D / cm^{-1}	-24.00	-22.40	-48.00	-41.50
E / D	0.229	0.256	0.192	0.246
D_{SS}	0.00	0.00	0.00	0.00
D_{SO}	-24.00	-22.40	-48.00	-41.50
D_Q	-5.60	-7.10	-5.30	-6.30
D_D	-18.40	-15.40	-42.70	-35.20

Supplementary Table 12. Pertinent geometrical parameters in 1 and 2 affected by applied external pressure. Bond lengths and angles are given in angstroms and in degrees, respectively. Average and optimized values are in parentheses and brackets, respectively.

**Pressure (GPa)**	d_{ax}(Re···X)[a]	d_{eq}(Re···X)[a]	d_{eq}(Re···N)	γ
Compound **1**				
0.0	2.331; 2.319<br>(2.325); [2.353]	2.299; 2.299<br>(2.299); [2.311]	2.084; 2.084<br>(2.084); [2.078]	86.6; 85.4<br>(86.0); [85.6]
4.3	2.307; 2.296<br>(2.302)	2.290; 2.290<br>(2.290)	2.011; 2.011<br>(2.011)	84.6; 84.4<br>(84.5)
Compound **2**				
0.0	2.492; 2.457<br>(2.475); [2.493]	2.438; 2.435<br>(2.436); [2.471]	2.164; 2.147<br>(2.155); [2.187]	85.2; 84.0<br>(84.6); [84.4]
4.3	2.424; 2.392<br>(2.408)	2.504; 2.416<br>(2.460)	2.103; 2.094<br>(2.098)	83.7; 81.7<br>(82.7)

[a]X = Cl or Br

Supplementary Table 13. **Comparison of the carbon-nitrogen and carbon-carbon bond lengths in the coordinated acetonitrile molecules in 1 at several applied pressures**. The bond lengths (in angstroms) are extracted from the crystal structure at each applied pressure, as described in the main text.

Pressure (GPa)	$\mathbf{d_{C\cdots N}}$	$\mathbf{d_{C\cdots C}}$
0.0	1.134	1.447
0.1	1.135	1.434
0.3	1.132	1.447
1.2	1.132	1.446
1.8	1.124	1.458
2.6	1.118	1.423
4.3	1.177	1.417

Supplementary Note 1

Prior to the high-pressure studies, dc magnetic susceptibility measurements were carried out at ambient pressure on a microcrystalline sample of **1** in the 2–300 K temperature range and different external magnetic fields (250 G and 0.1 T). At ambient pressure and temperature, the $\chi_M T$ value is 1.59 $cm^3\ mol^{-1}$ K, a value expected for a magnetically isolated mononuclear Re^{IV} complex (S = 3/2, g = 1.8-1.9). Upon cooling, this value continuously decreases, first smoothly in the high temperature region and then sharply down to 150 K, to reach a minimum at *ca*. 6.5 K. At lower temperatures, $\chi_M T$ exhibits an abrupt increase and finally decreases at the lowest temperatures (see Supplementary Figure 5). The increase is more pronounced at lower external dc magnetic fields where saturation effects are minimized. The variation of $\chi_M T$ in the high-temperature range for **1** reveals an antiferromagnetic exchange between Re^{IV} ions. Moreover, the abrupt increase of $\chi_M T$ in the very low-temperature domain can be attributed to spin canting, that is, non-linearity of the individual spins aligned in an anti-parallel fashion by means of intermolecular antiferromagnetic coupling, which has been observed in previously studied Re^{IV} complexes. The large anisotropy of the Re^{IV} ion accounts for this phenomenon. The magnetic susceptibility data was analysed through the spin-Hamiltonian of eqn (1)

$$\hat{H} = D\left[\hat{S}_z^2 - S(S+1)/3\right] + g\beta\left(H_z\hat{S}_z + H_x\hat{S}_x + H_y\hat{S}_y\right) \quad (1)$$

where the first term accounts for the zero-field splitting (*zfs*) of the Re^{IV} ion and the last term is the Zeeman effect. In order to avoid over-parameterisation, an isotropic g factor was assumed. The best-fit parameters for **1** at ambient pressure are $|D_{EXP}|$ = 37.1 cm^{-1}, g = 1.81, and $R = 2.4 \times 10^{-5}$ {R is the agreement factor defined as $\Sigma[\chi_M T_{obs}(i) - \chi_M T_{calc}(i)]^2 / \Sigma[\chi_M T_{obs}(i)]^2$}. The calculated curve reproduces the experimental data very well in the temperature range 20–300 K (Supplementary Figure 5).

The field-cooled magnetisation of **1** at 250 G (see inset in Supplementary Figure 5) shows the occurrence of magnetic order below 6.7 K, confirming the long-range ordering of the canted antiferromagnet. Indeed, variable-field magnetisation data collected at 2.0 K between −7 and 7 T reveals complete reversibility of the magnetisation (Supplementary Figure 6) but without showing any evidence of saturation at the highest magnetic field available; this last feature being typical of weak ferromagnets. A hysteresis loop is observed at 2.0 K with values of coercive field (H_c) and remnant magnetisation (M_r) of 850 G and 0.068 μ_B, respectively. The evaluation of the canting angle (α) can be performed through eqn (2)

$$\sin(\alpha/2) = M_w/(2M_s) \quad (2)$$

where M_w is the magnetisation induced by a very weak magnetic field, and M_s is the saturation magnetisation. Although no saturation is reached at 7 T, a value of 2.0º can be estimated for **1**, by assuming extrapolated values of M_s and $M_w \approx M_\mathrm{r}$ at 2.0 K.

Supplementary Note 2

The pertinent geometrical parameters in **1** and **2** that significantly change with pressure are shown in Supplementary Table 12 and Supplementary Figures 12 and 13. A theoretical analysis on how each parameter changes the *zfs* was carried out on ideal models of **1** and **2** at pressures of 0.0 GPa and 4.3 GPa. From these results, several conclusions can be drawn: i) Similar trends were found from both PBE-DFT and CAS calculations. ii) The values of the studied structural parameters for the more stable molecular geometry are close to those observed in the experimental ambient pressure structure. Slightly longer metal-ligand bond lengths are observed in some cases because calculations were performed in the gas phase where more relaxed molecular geometries are expected, and because DFT methods often overestimate the bond lengths. iii) The changes in energy from the molecular geometries at 0.0 GPa to those at 4.3 GPa are very small indeed - less than 3 kcal/mol - which supports the fact that geometrical changes induced by external pressure are rather easy. iv) The effect of each geometrical change on the *zfs* is qualitatively equivalent in **1** and **2**, with the exception of the Re-N equatorial distance [d_{eq}(Re···N)] and the N-Re-N bite angle. Indeed the change in the d_{eq}(Re···N) parameter with pressure leads to an increasing *D* in **2**, but to a decreasing *D* in **1**, as observed experimentally.

Supplementary Methods

Computational methodology. To estimate the nature and magnitude of the intermolecular magnetic exchange interactions in **1** and **2**, calculations were performed with the Gaussian09 package using the CAM-B3LYP functional (a long range corrected version of B3LYP) and the quadratic convergence approach[1-5]. Double-ζ and Los Alamos effective core potentials, as proposed by Hay and Wadt, were used for the Re^{IV}, Cl^{-} and Br^{-} ions[6-8]. Ahlrichs double-ζ basis set was used for the remaining atoms[9]. Two-electron integrals and their derivatives were computed from Douglas-Kroll-Hess (DKH) 2nd order scalar relativistic calculations[10,11]. An approach based on the use of broken-symmetry (BS) functions built from localised orbitals was used to evaluate the energies of several spin states[12]. The BS functions, which provide positive or negative spin densities on the paramagnetic centres, were obtained from the guess functions generated with the fragment tool implemented in Gaussian09. The full experimental geometries were used for all calculated complexes, which are shown in Supplementary Figures 7 and 8. Parameters corresponding to the acetonitrile solvent were included to simulate the electronic effects of the surrounding molecules[13]. However, contrary to what is found in systems with other metal ions, the intermolecular magnetic interactions calculated in **1** and **2** in the gas phase are similar to those found using a solvent model (Supplementary Table 10).

Calculations of the *zfs* parameters were performed with version 3.0 of the ORCA program[14]. The TZVP basis set proposed by Ahlrichs, and tight SCF criteria were used in all cases[9]. Relativistic effects for the Re^{IV} ion were introduced from a zero-order regular approximation (ZORA)[15]. For complete active space (CAS) calculations, this auxiliary basis set was replaced by TZV/C[16-18]. Experimental geometries of **1** and **2** were used in the theoretical calculations. The *zfs* parameters were evaluated from CAS and N-Electron Valence State Perturbation Theory (NEVPT2) calculations by including contributions from ten quartet and twenty doublet states generated from electron promotion between *d* orbitals, which corresponds to the full active space built from only the five *d* orbitals of the Re^{IV} ion[19-21].

Molecular dynamics simulations were performed with version 3.2 of the SIESTA[22] program starting from an optimised structure. A cell with four $[ReCl_4(MeCN)_2]$ molecules was considered where the symmetry was removed, allowing freedom for molecular reorganisation. The disorder of the acetonitrile molecules was removed, cleaning those atomic positions that were duplicated. The GGA exchange-correlation functional proposed by Perdew, Burke and Erzernhof (PBE)[23] with a DZP basis set was employed for the external electrons and pseudopotentials generated following the approach

proposed by Trouiller and Martins for the internal electrons[24]. The core radii for the s, p and d, and f components of the Re^{IV} ions are 2.67, 2.74 and 2.51 Å, respectively. The cut-off radii were 1.48, 1.25 and 1.33 Å for N, C and H atoms, respectively, and 1.66 and 1.88 for the s and p, and d and f orbitals of the Cl^- ions. For the Br^- ions, the cut-off radii were 1.84, 2.19, 1.68 and 2.30 for the s, p, d and f orbitals, respectively. Values of 50 meV for the energy shift, 400 Ry for mesh cut-off and 5.0 Å for the k-grid cut-off were used. Atmospheric pressure and T = 5 K, which were controlled by Parrinello-Rahman[25] and Nose[26] methods, respectively, were considered during the simulation employing 1000 steps at a rate of 1fs per step. In order to generate the distribution diagrams (Figure 5 and Supplementary Figure 11), each one of the calculations during the dynamics simulation is considered one event.

Supplementary References

1. Frisch, M. J. *et al*. Gaussian 09 (Revision C.01) ed.; Gaussian, Inc: Wallingford CT, 2009.
2. Becke, A. D. Density-Functional Exchange-Energy Approximation with Correct Asymptotic Behavior. *Phys. Rev. A* **38**, 3098-3100 (1988).
3. Becke, A. D. Density-Functional Thermochemistry. III. The Role of Exact Exchange. *J. Chem. Phys*., **98**, 5648-5652 (1993).
4. Lee, C. T. *et al*. Development of the Colic-Salvetti correlation-energy formula into a functional of the electron density. *Phys. Rev. B* **37**, 785-789 (1988).
5. Yania, T., Tew, D. P., & Handy, N. C. A new hybrid exchange–correlation functional using the Coulomb-attenuating method (CAM-B3LYP). *Chem. Phys. Lett*., **393**, 51-57 (2004). Schäfer, A., Horn, H. & Ahlrichs, R. Fully optimized contracted Gaussian-basis sets for atoms Li to Kr. *J. Chem. Phys.*, **97**, 2571-2577 (1992).
6. Hay, P. J. & Wadt, W. R. Ab initio effective core potentials for molecular calculations. Potentials for the transition metal atoms Sc to Hg. *J. Chem. Phys*., **82**, 270-283 (1985).
7. Wadt, W. R. & Hay, P. J. Ab initio effective core potentials for molecular calculations. Potentials for main group elements Na to Bi. *J. Chem. Phys*., **82**, 284-298 (1985).
8. Hay, P. J. & Wadt, W. R. Ab initio effective core potentials for molecular calculations. Potentials for K to Au including the outermost core orbitals. *J. Chem. Phys*., **82**, 299-310 (1985).
9. Schäfer, A., Huber, C. & Ahlrichs, R. Fully optimized contracted Gaussian basis sets of triple zeta valence quality for atoms Li to Kr. *J. Chem. Phys.* **100**, 5829-5835 (1994).
10. Douglas, M. & Kroll, N. M. Quantum electrodynamical corrections to the fine structure of helium Original. *Ann. Phys*. **82**, 89-155 (1974).
11. Hess, B. A. Applicability of the no-pair equation with free-particle projection operators to atomic and molecular structure calculations. *Phys. Rev. A*, **32**, 756-763 (1985).
12. Ruiz, E. *et al*. Broken symmetry approach to calculation of exchange coupling constants for homobinuclear and heterobinuclear transition metal complexes. *J. Comput. Chem*., **20**, 1391-1400 (1999).
13. Tomasi, J., Mennucci, B. & Cancès, E. The IEF version of the PCM solvation method: an overview of a new method addressed to study molecular solutes at the QM ab initio level. *J. Mol. Struct. (THEOCHEM)* **464**, 211-226 (1999).

14. Neese, F. The ORCA program system. *WIREs Comput. Mol. Sci.*, **2**, 73-78 (2012).
15. Chang, Ch., Pelissier, M. & Durand, Ph. Regular Two-Component Pauli-Like Effective Hamiltonians in Dirac Theory. *Phys. Scr.* **34**, 394-404 (1986).
16. Eichkorn, K., Treutler, O., Ohm, H., Haser, M. & Ahlrichs, R. Auxiliary basis sets to approximate Coulomb potentials. *Chem. Phys. Lett.* **240**, 283-290 (1995).
17. Eichkorn, K., Treutler, O., Ohm, H., Haser, M. & Ahlrichs, R. Auxiliary basis sets to approximate Coulomb potentials *Chem. Phys. Lett.* 2**42**, 652-660 (1995).
18. Eichkorn, K., Weigend, F., Treutler, O. & Ahlrichs, R. Auxiliary basis sets for main row atoms and transition metals and their use to approximate Coulomb potentials. *Theor. Chem. Acc.* **97**, 119-124 (1997).
19. Angeli, C., Cimiraglia, R., Evangelisti, S., Leininger, T. & Malrieu, J.-P. Introduction of *n*-electron valence states for multireference perturbation theory. *J. Chem. Phys*. **114**, 10252-10264 (2001).
20. Angeli, C., Cimiraglia, R. & Malrieu, J.-P. *N*-electron valence state perturbation theory: a fast implementation of the strongly contracted variant. *Chem. Phys. Lett*. **350**, 297-305 (2001).
21. Angeli, C., Cimiraglia, R. & Malrieu, J.-P. *N*-electron valence state perturbation theory: A spinless formulation and an efficient implementation of the strongly contracted and of the partially contracted variants *J. Chem. Phys*. **117**, 9138-9153 (2002).
22. Artacho, E. *et al*. SIESTA 1.3, 2001.
23. Perdew, J. P., Burke, K. & Ernzerhof, M. Generalized Gradient Approximation Made Simple. *Phys. Rev. Lett*. **77**, 3865-3868 (1996).
24. Troullier, N. & Martins, J. L. Efficient pseudopotentials for plane-wave calculations. *Phys. Rev. B: Condens. Matter* **43**, 1993-2006 (1991).
25. Parrinello, M. & Rahman, A. Crystal Structure and Pair Potentials: A Molecular-Dynamics Study. *Phys. Rev. Lett*., **45**, 1196-1199 (1980).
26. Nosé, S. A unified formulation of the constant temperature molecular-dynamics methods. *J. Chem. Phys*. **81**, 511-519 (1984).
